# Discernible interindividual patterns of global efficiency decline during theoretical brain surgery

**DOI:** 10.1038/s41598-024-64845-4

**Published:** 2024-06-25

**Authors:** Lin Yueh-Hsin, Nicholas B. Dadario, Si Jie Tang, Lewis Crawford, Onur Tanglay, Hsu-Kang Dow, Isabella Young, Syed Ali Ahsan, Stephane Doyen, Michael E. Sughrue

**Affiliations:** 1 Centre for Minimally Invasive Neurosurgery, Prince of Wales Private Hospital, Suite 19, Level 7 Prince of Wales Private Hospital, Randwick, Sydney, NSW 2031 Australia; 2 Robert Wood Johnson Medical School, Rutgers University, 125 Paterson St, New Brunswick, NJ 08901 USA; 3 School of Medicine, 21772 University of California Davis Medical Center, 2315 Stockton Blvd., Sacramento, CA 95817 USA; 4 Omniscient Neurotechnology, Level 10/580 George Street, Sydney, NSW 2000 Australia; 5 School of Computer Science and Engineering, University of New South Wales (UNSW), Building K17, Sydney, NSW 2052 USA; 6 Centre for Minimally Invasive Neurosurgery, Prince of Wales Private Hospital, Suite 3, Level 7 Barker St, Randwick, NSW 2031 USA

**Keywords:** Neurosurgery, Percolation theory, Connectome, Graph theory, Global efficiency, Tumor, Structural network, Neuroscience, Anatomy

## Abstract

The concept of functional localization within the brain and the associated risk of resecting these areas during removal of infiltrating tumors, such as diffuse gliomas, are well established in neurosurgery. Global efficiency (GE) is a graph theory concept that can be used to simulate connectome disruption following tumor resection. Structural connectivity graphs were created from diffusion tractography obtained from the brains of 80 healthy adults. These graphs were then used to simulate parcellation resection in every gross anatomical region of the cerebrum by identifying every possible combination of adjacent nodes in a graph and then measuring the drop in GE following nodal deletion. Progressive removal of brain parcellations led to patterns of GE decline that were reasonably predictable but had inter-subject differences. Additionally, as expected, there were deletion of some nodes that were worse than others. However, in each lobe examined in every subject, some deletion combinations were worse for GE than removing a greater number of nodes in a different region of the brain. Among certain patients, patterns of common nodes which exhibited worst GE upon removal were identified as “connectotypes”. Given some evidence in the literature linking GE to certain aspects of neuro-cognitive abilities, investigating these connectotypes could potentially mitigate the impact of brain surgery on cognition.

## Introduction

Surgery for brain tumors, specifically diffuse gliomas (WHO grade 2–4), involves removing a part of the cerebrum, often a quite large part of a lobe of the hemisphere. While the survival benefits of this intervention have been documented, the rates of cognitive and emotional morbidity related to aggressive neuro-oncology surgery have also been described, and there is a poor understanding of how to avoid or minimize their severity^1–4^. Generally the focus in neurosurgical oncology has been aimed at identifying and avoiding the removal of so called “eloquent” regions which caused obvious speech or motor deficits when resected^5^. While these strategies were wise, the idea that the rest of the brain is “non-eloquent” and thus can be removed with impunity, has only been justified by our lack of understanding about how to avoid these deficits^6–9^.

While one possible approach to reducing the cognitive footprint of brain surgery^10^ is to identify additional sites which should not be violated, such as large-scale brain networks like the default mode and salience networks, it has become increasingly clear that at least some of the cognitive capacity of the brain arises from more complex multi-network interactions and global topological characteristics^11–17^. For example, some data suggest that global measures of network organization, such a global efficiency (GE) or pathlength, relate closely to cross modal measures of intelligence^18,19^, albeit there are other studies which do not see such an association^20^ or observe this only in a select population of individuals^19^. Lowered brain network efficiency is associated with impairments in memory, executive function, and processing speed among older healthy individuals^21^ or with depression^22^, dementia^23^, autism^24^ and mild cognitive impairments^25^. In particular, GE is one graphic theory metric of network efficiency which has been well-studied in the context of altered cognitive outcomes in a number of brain disorders, such as Alzheimer’s disease^26^, traumatic brain injury^27^, and small-vessel disease^28^. Therefore, GE provides a plausible metric for neurosurgeons to understand how tolerable a network is to lesions induced during surgery and the subsequent risk for cognitive morbidity^18,19^. Nevertheless, the ability to pre-operatively predict and understand the neurologic consequences of transgressing certain brain regions –and importantly, how these regions differ between certain individuals—is a uniquely neurosurgical problem requiring novel experimentation. It has been known for some time that large parts of certain lobes of the brain can be removed without drastic consequence. However, it is unlikely that there are no limits or consequences to increasingly extreme resections. While we know the brain connectome employs a degree of redundancy, the brain is not infinitely able to tolerate progressive damage, and there are clearly lines we cannot cross without causing a loss of function. Given the complex interactions which could not be accounted for by simple anatomic guidelines, it seems unlikely that this problem could be reduced to a simple set of rules for regions that cannot undergo resection.

To understand possible post-operative cognitive deficits following specific surgical decisions, we utilized graph theory measures of GE as a proxy for a patient’s ability to integrate information efficiently and therefore their post-operative cognitive functioning. A simulation of clinically plausible resections of regions of the brain delineated into parcellations in a cohort of healthy adults was used to determine the effect of these interventions on GE. Specifically, an optimal percolation theory^29^ approach was applied on structural cortical networks constructed from diffusion tractography of diffusion tensor images (DTI) using a highly anatomically specific parcellation scheme^30^ in order to investigate a location-specific pattern of injury as identified by a structural brain connectivity measure that is predictive of patient cognitive functioning. We found that while some brain regions were no more detrimental to remove than others, the exact parcellation with the worst GE was not the same across the cohort. Nevertheless, there were distinct patterns of regions that tended to be among the worst to remove. We termed these patterns “connectotypes,” with the obvious implication that given the epicenters of these connectotypes are not physically adjacent to each other, knowing a subject’s connectotype could provide clinically relevant information for decision-making and neurosurgical planning.

## Methods

### Dataset

Magnetic resonance images (MRI) consisting of diffusion-weighted and T_1_-weighted data of eighty healthy subjects were randomly selected from OpenNeuro (https://openneuro.org). Participant IDs were provided in Supplementary Table 1.

### Image preparation

A standard processing of steps was employed for pre-processing and processing the diffusion tractography data using the Dipy package^31^. This method of analysis has been previously described^32–34^ but will be included in brief: (1) the diffusion images were resliced to ensure isotropic voxels, (2) motion correction was performed using a rigid body alignment (with 6 degrees of freedom), slices with excess movement (defined as the root means squared of the temporal change of a voxel (DVARS) > 2 sigma from the mean slice) were eliminated^33,34^, (3) the signal was represented using spherical harmonics, and multiplerotation invariant spherical harmonic features were computed to estimate a region- and tissue-specific linear mapping between the signal from different sites^35^, (4) the T_1_ images were skull stripped using a convolutional neural net (CNN) which were then inverted and aligned to the DT image using a rigid alignment and then used as a mask to skull strip the DT, (5) gradient distortion correction was performed using a diffeomorphic warping method which aimed to locally equalize the DT and T_1_ images, (6) eddy current correction was performed, (7) Fiber response function was estimated and the diffusion tensors were calculated using constrained spherical deconvolution (peaks were extracted using the EuDX algorithm with a relative peak threshold of 0.5 and minimum separation angle of 20°), and (8) deterministic tractography was performed with random seeding of four fibers per voxel seeded evenly throughout white matter voxels with a fractional anisotropy (FA) value > 0.3, creating about 300,000 streamlines per brain. Subjects whose scans contained artifacts (such as brain distortions, head motion, and venetian blind artifacts), poor field of view, inadequate slices, and other general faults in the scans were removed from the dataset.

### Machine learning-based parcellation of the cerebral cortex

The images were further processed into region of interests (ROIs) by using the Omniscient Neurotechnology software (https://www.o8t.com). Omniscient software is a machine learning-based, subject-specific version of the Human Connectome Project (HCP) Multi-Model Parcellation version 1.0 brain atlas^30^. The general concept is to use a machine learning-based technique for parcellating a brain based on structural connectivity, avoiding the limitations of assigning brain voxels and anatomic-based methods using spatial positioning. The Omniscient software used to create brain parcellations for the 80 subjects in our study was trained using the subject specific version of the HCP atlas based on diffusion tractography structural connectivity as described previously^7,32,34^. This method was created by training a model on 200 normal subjects by processing T_1_ and DT images as described in the section above. An HCP atlas in NIFTI MNI space and the 19 subcortical components (8 subcortical structures in each hemisphere, 1 cerebellar region for each hemisphere, and one brain stem region) from the Freesurfer software (https://surfer.nmr.mgh.harvard.edu/fswiki/FreeSurferVersion3) were then warped onto each brain. The structural connectivity was calculated between every pair of this atlas and a set of Regions of Interest (ROIs) based on the streamlines which terminated within an ROI. This step both allowed for the generation of feature vectors (a voxel to parcellation structural connectivity matrix) and the generation of a centroid of the parcellation which was utilized to constrain the voxels studied for assignment of a given parcellation to a plausible area in the vicinity of its typical position. The feature vectors for each region were then used as a training set to fit a gradient boosted tree-based model using XGBoost. We used XGBoost for three reasons. (1) It was computationally effective—the X in the algorithm stands for Extreme and relates to the highly parallelised approach of this algorithm. (2) It delt with many different types of data, in this case non-linear and discrete observations (number of tracts between parcellation x and y). (3) Finally, it provided the best performance on training with tenfold cross validation. To ensure clinical relevance of the model, we optimized the algorithm based on precision.

This Omniscient model was then used to classify specific subject parcellations by warping the HCP atlas to the new brains of the 80 patients obtained from the OpenNeuro data and collecting a set of feature vectors of the connectivity of each voxel based on this first pass. The feature vectors were then used to determine each voxel parcellation identity. This method transformed an atlas onto a brain and calculated the structural connectivity between every pair of the applied atlas and a set of ROIs, allowing for the generation of feature vectors and parcellation centroid to determine each voxel parcellation identity.

### Creation of theoretical region resections

With the parcellations mapped by the Omniscient algorithm, a structural connectivity graph was created for each subject with each parcellation as nodes and the number of streamlines between the nodes representing the edges. To limit our analysis to performing only anatomically plausible brain surgeries, a weighted undirected graph of physical adjacency in the HCP atlas was created where all cortical parcellations that shared a physical border had an edge in the graph. We then created a list of all connected components in this graph which represented possible brain resection where a series of parcellations adjacent to each other were included. Specifically, the adjacency relation formed an undirected graph *G* = *(V, E)* where vertices *V* were the collection of the parcellations and edges *E* were the adjacency relation between each parcellation. Then, the depth-first search (DFS)^36^ traversal of G was performed to generate all deletion permutations from one parcellation, deleting up to ten parcellations in a series. We limited each deletion to a specific brain lobe or region to keep the theoretical resections clinically plausible as multi-lobar resections are rare in clinical practice. Ultimately, 8 distinct cortical regions were assessed. This included the frontal lobe, sensorimotor cortex, medial frontal lobe, operculum, temporal lobe, lateral parietal lobe, medial parietal lobe, and occipital lobe.

### Measuring injury patterns with global efficiency

To study the impact of location-specific patterns on injury, we employed the structural connectivity measure of GE. GE can be defined as the inverse of the average characteristic path length between all nodes in the network and is understood as a measure for network’s capacity to transfer information^37^. Formally, GE is calculated as,

$${E}_{global(i)}=\frac{1}{{N}_{\left(i\right)}\left({N}_{\left(i\right)}-1\right)}\sum_{\begin{array}{c}j,k\in {N}_{\left(i\right)}\\ j\ne k\end{array}}\frac{1}{\begin{array}{c}{L}_{j,k (i)}\\ \end{array}}.$$

First, we calculated the GE for the entire brain. Then for each combination of plausible brain surgery, we set the edges for all the nodes in that combination to 0 which removed it from the graph. We then recalculated the GE for the new graph to determine the effect of that deletion.

The next step was to calculate how each deletion combination progressed between individuals to find the epicenters and core parcellations which conferred the greatest consequences on a network’s GE. The GE value for each combination was sorted in descending order to create a list *L*. Furthermore, $${S}_{n}$$ represented a set of all the combinations with *n* number of parcellations. Therefore $${S}_{1}$$ represented the sets containing all combinations with one parcellation. In the sorted list *L*, if all the combinations with one parcellation were not mixed with combinations of two parcellations in the sorted list, then we labeled Step 1 to 2 as stepwise. If all Step $${N}_{i}$$ to $${N}_{i+1}$$ were satisfied as stepwise, the patient was qualified as a perfect stepwise case. If the top three Steps (Step1 to 2, Step 2 to 3, Step 3 to 4) were stepwise but not the rest, the patient was qualified as a partial-stepwise case. If none of the Step $${N}_{i}$$ to $${N}_{i+1}$$ were stepwise, the patient qualified as a non-step case. GE of each percolation result was then arranged ascendingly, and the result was quantified across eighty individuals. A program was written in Python to identify the distribution of percolation combination to show the worst combination in descending order from $${S}_{1}, {S}_{2}\dots {S}_{i}$$. The most frequently occurring worst parcellation among the eighty subjects, after percolating combinations in $${S}_{1}$$, was identified as the epicenter of the lobe. Individuals were singled out if they had a different parcellation identified as the worst area for resection after percolation at $${S}_{1}$$. Among those individuals, the worst combinations of each set were selected for further analysis. For a specific epicenter of interest, a program was written to find in the list *L* when $${S}_{i}$$ epicenter would show up ($${S}_{r})$$ in that subpopulation of individuals Then, we further explored the expected minimal set ($${S}_{m})$$ between epicenter and the worst parcellation after percolation in thoseindividuals. The difference (D) was calculated as $${S}_{r}-{S}_{m}$$ which a more positive D beingdirectly proportional to the closeness between the selected parcellation and epicenter.

All calculations were performed in Python using the Pandas^38^, Numpy^39^, NetworkX^40^ and iGraph^41^ Packages.

### Ranking parcellations by their centrality

PageRank (PR) of each parcellation was determined as previously described^42^. PR is an algorithm that measures the transitive influence or connectivity of nodes, which is calculated as:

$$PR\left({P}_{i}\right)=\frac{(d)}{n}+\left(1-d\right)\times \sum_{l\_(j,i)\in E}\frac{PR({P}_{j})}{Outdegree({P}_{j})}.$$

PR score ranged from 1 to 379, with higher score predicting the degree of eloquence. PR results were ranked among all the parcellations per hemisphere, with 1 as the highest in PR. The ranking between PR and GE were compared at single deletion level.

## Results

### Series of simulated brain surgeries demonstrate a location-specific pattern of injury

To determine which surgeries most adversely affected GE, an algorithm was created to perform all clinically plausible brain resections defined as cortical parcellations in direct continuity with other regions in a group (Supplementary Information Section I). In this way, we were not studying every possible combination since removing multiple lobes or combinations of regions that were not next to each other is very uncommon in practice.

The idea that specific nodes would have a greater deleterious effect than others has previously been demonstrated^29,43,44^. We first evaluated whether the drop in GE after percolation would be solely determined by how many parcellations of the brain were removed, or whether some specific regions would be worse than others in a way which is out of proportion to the number of total brain regions removed. We found that in every lobe for each subject, that there were parcellations which when removed lead to a worse GE than many combinations which removed more regions (Supplementary Information Section II).

**Figure 1:**
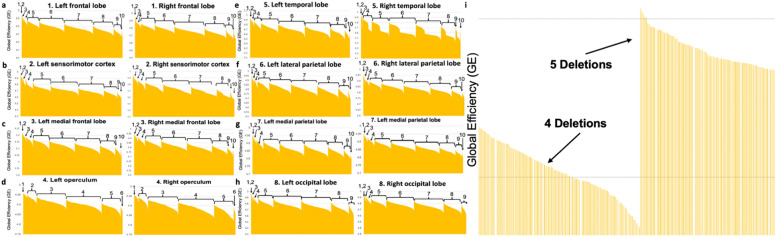
Serial nodal removals were performed from graphs as one possible surgical combination. GE was calculated after each nodal removal. The calculated GE (y-axis) was then grouped and graphed based on the number of nodal removals performed (numbers above the graph). Panels (**a**–**h**) display this analysis within each lobe. Panel (**i**) is an example magnification of panels (**a**–**h**). There is no x-axis for panels (**a**–**i**) since the x-axes are made up of many small strips of bar (with one strip of bar representing one possible surgical combination).

To investigate a possible location-specific pattern of injury, we carried out serial combinations of nodal deletions (of up to ten serial deletions) of the graph. This was done across the 8 major brain lobes. We found that each site of deletion comprised a unique decrease in GE across all cortical regions. It showed a “non-stepwise” pattern in GE drop, which demonstrated removal of smaller regions could be more damaging than removing a bigger region (Fig. 1a–h). This “non-stepwise” pattern further demonstrated the loss in GE could not only be explained by the *number* of parcellations removed in all cases. This is shown in Fig. 1i, which is a magnification of the panels a-h in Fig. 1, where some parcellations with 4 nodal removals can have worse GE than some parcellations with 5 nodal removals. Specifically, each site of deletion presented a “non-stepwise” pattern in GE drop such that a smaller number of deletions (i.e. 4 parcellations) would constitute a worse outcome compared a larger number of deletions (i.e. 5 parcellations) as shown in panel I. These data illustrate that lowered GE and patient cognitive functioning may not be entirely explainable by just the amount of cortex removed. Instead, neurosurgical damage to highly specific regions may induce disproportionally worse cognitive damage than just removing more cortex.

### Surgical tolerance can only be partly explained by hubness

Given that there were specific deletions which were disproportionately worse than larger deletions consisting of more areas, we evaluated two possible hypothetical phenomena which could occur at larger deletions: either (1) there were specific combinations which would emerge when larger deletions hit specific collateral pathways that were uniquely worse than those seen at smaller deletions, or (2) the worst large deletions were just combinations consisting of smaller deletions with low GE. First, the most common worst deletions among the subjects (regardless of whether they were the worst single deletion in that person) were determined (Table 1). However, in some individuals, we observed they had other specific deletions that were different from the others, thus requiring further examination as described in the next section. Secondly, as observed in all the lobes, larger deletion sizes, often contained previous worse deletions from smaller deletion sizes. For example, the worst combinations of 7 or 8 would include the component of the previous deletion. We provide an example of this in the frontal lobe (Table 2). Additional results related to each individual lobe are presented in the Supplementary Material Tables S1–S10.

**Table 1:** The top worst areas in each cortical region per cerebral hemisphere.

	Worst single deletion in the left hemisphere	Worst single deletion in the right hemisphere
1. Frontal lobe	L_45 (n = 29/80; 36%)	R_8BL (n = 21/80; 26%)
	L_10d (n = 15/80; 19%)	R_8AV (n = 16/80; 20%)
	L_8AV (n = 14/80; 18%)	R_44 (n = 12/80; 15%)
		R_45 (n = 11/80; 14%)
2. Sensorimotor cortex	L_SFL (n = 48/80; 60%)	R_SFL (n = 30/80; 38%)
	L_6ma (n = 12/80; 15%)	R_4 (n = 16/80; 20%)
		R_6ma (n = 13/80; 16%)
3. Medial frontal lobe	L_9m (n = 31/80; 39%)	R_9m (n = 33/80; 41%)
	L_SCEF (n = 24/80; 30%)	R_10v (n = 14/80; 18%)
	L_24dd (n = 13/80; 16%)	R_SCEF (n = 11/80; 14%)
4. Operculum	L_A4 (n = 54/80; 68%)	R_A4 (n = 41/80; 51%)
	L_PoI2 (n = 9/80; 11%)	R_43 (n = 13/80; 16%)
		R_PoI2 (n = 12/80; 15%)
5. Temporal lobe	L_TGd (n = 43/80; 54%)	R_TGd (n = 52/80; 65%)
	L_TE1p (n = 27/80; 34%)	R_TE1p (n = 8/80; 10%)
		R_PHT (n = 7/80; 9%)
		R_TE2a (n = 6/80; 8%)
6. Lateral parietal lobe	L_7AM (n = 40/80; 50%)	R_PFm (n = 29/80; 36%)
	L_PFm (n = 13/80; 16%)	R_7AM (n = 15/80; 19%)
		R_PGp (n = 14/80; 18%)
		R_7PC (n = 10/80; 13%)
7. Medial parietal lobe	L_POS2 (n = 39/80; 49%)	R_POS2 (n = 42/80; 53%)
	L_7m (n = 11/80; 14%)	R_PCV (n = 14/80; 18%)
		R_POS1 (n = 13/80; 16%)
8. Occipital lobe	L_FFC (n = 31/80; 39%)	R_V1 (n = 24/80; 30%)
	L_V2 (n = 21/80; 26%)	R_FFC (n = 17/80; 21%)
	L_V3 (n = 14/80; 18%)	R_V3 (n = 16/80; 20%)
		R_V2 (n = 12/80; 15%)

There was a large amount of similarity in which the worst deletions were across a large fraction of subjects. This was found regardless of whether they were the worst single deletion in a single person. Importantly, a subset of individuals demonstrated that other specific deletions were the worst compared to the majority of individuals.

**Table 2:**
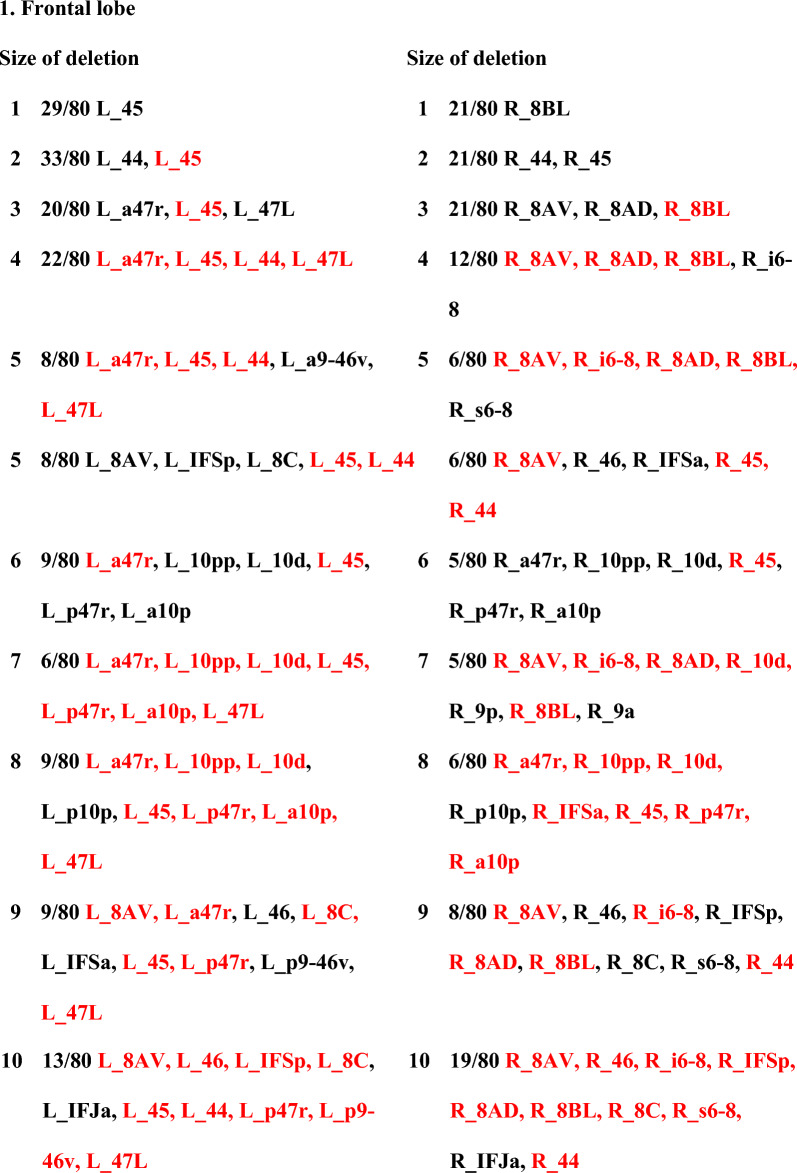
List of the worst parcellation combinations deleted among 80 individuals in the frontal lobe.

Parcellations that are highlighted in red mean the occurrence of the worst deletion in previous combination. Progression of deleted combination has shown to be an accumulation of previous worst areas in percolation.

It has been demonstrated that the deletion of hubs from a graph has a disproportionately deleterious effect on the efficiency of a graph^29,43,44^. Given that specific nodal deletion seemed to explain the worst deletions for both smaller and larger theoretical surgeries, we tested the hypothesis whether a measure of hubness, such as PageRank centrality^7^, could explain which nodes cause the worst effect on GE when deleted. Our results supported this hypothesis to an extent: across eight cortical regions, the frequency that the parcellation with the highest PageRank centrality in a lobe predicted the worst deletions in that lobe ranged from 35 to 75% in different subjects.

Presently, neurosurgical interventions rarely can limit the damage to individual parcellations. Therefore, we further addressed the possibility that minor changes in the border of parcellations might have led to changes in centrality in neighboring parcellations, which might lead to clinically irrelevant differences for resective brain surgery. The node with the highest page rank centrality in a lobe’s top 3 worst deletions ranged from 83 to 98% in both left and right hemispheres, suggesting that knowing the node with the highest centrality was useful but not always deterministic for evaluating the worst deletions (Table 3).

**Table 3:** A summary of the successful rate by using PageRank (PR) to predict the worst area for deletion, its neighbouring parcellation and the top 3 worst areas for deletion.

	Left hemisphere			Right hemisphere		
	PageRank predicts			PageRank predicts		
	The worst deletion	The worst deletion and its neighbouring deletion	Top 3 worst deleted area	The worst deletion	The worst deletion and its neighbouring deletion	Top 3 worst deleted area
1. Frontal lobe	53/80; 66%	61/80; 76%	69/80; 86%	57/80; 71%	57/80; 71%	74/80; 93%
2. Sensorimotor cortex	57/80; 71%	66/80; 83%	78/80; 98%	55/80; 69%	66/80; 83%	75/80; 94%
3. Medial frontal lobe	27/80; 34%	31/80; 39%	68/80; 85%	51/80; 64%	57/80; 71%	66/80; 83%
4. Operculum	45/80; 56%	63/80; 79%	66/80; 83%	53/80; 66%	68/80; 85%	74/80; 93%
5. Temporal lobe	56/80; 70%	59/80; 74%	78/80; 98%	52/80; 65%	60/80; 75%	76/80; 95%
6. Lateral parietal lobe	52/80; 65%	59/80; 74%	72/80; 90%	44/80; 55%	51/80; 64%	76/80; 95%
7. Medial parietal lobe	60/80; 75%	77/80; 96%	74/80; 93%	31/80; 39%	51/80; 64%	73/80; 91%
8. Occipital lobe	40/80; 50%	58/80; 73%	68/80; 85%	41/80; 51%	63/80; 79%	66/80; 83%

### There is evidence for patient specific “connectotypes” in differential surgical tolerance

Knowing the cortical region with the highest centrality in a lobe was useful for determining the worst deletions in this lobe, but it was not commonly the case that the hub of a lobe was the worst area. Therefore, we next evaluated if there was a discernible pattern which would determine the worst surgery we could perform in each lobe. We noted two key insights from a closer analysis of the patterns of worst injuries. First, specific parcellations seemed to be part of the worst deletion combinations in a large fraction of subjects, regardless of whether they were the top worst single deletion in that person. Second, we noted that the worst large deletions in a lobe were largely a progression of additional parcellations added onto the worst (Table 2).

We then hypothesized the following: first, that there are certain regions, regardless of which specific single parcellation was the worst single region to remove, would show up early in the progression of worst deletions regardless of where it started, and second, that there would be distinct patterns by which some subjects would differ in their progression which may explain why some subjects differ from others. We termed this possible fundamental difference in connectivity thatled to differential tolerance a “connectotype,” implying that there was something fundamentally different between two individuals leading to differences in the progression of worst deletions at each extent of deletion.

To assess the possibility that such connectotypes might exist, we explored the possibility that each connectotype would have an “epicenter” region which would arise in the list of worst deletions earlier than expected based on the identity of the worst parcellation in that subject’s progression sequence. In other words, if there were not epicenters which defined specific patterns of sensitivity, then no specific parcellation would show up earlier than expected in the progression sequence based on its physical distance from the worst region in that person’s lobe. Importantly, note that because our algorithm only allowed for deletions of combinations of parcellations which specifically form a connected component, if parcellation x is at closest 3 parcellations away from parcellation y, then the earliest we would expect to see parcellation y in a sequence of worst deletions in a subject for whom the worst single deletion was parcellation x would be as a member of the worst deletion among deletion combinations involving 4 parcellations. Falsification of this null hypothesis would be to find parcellation y arising in the worst combinations of 2 or 3 deletions because this would imply that this region was important regardless of who was the worst single parcellation and that it was not merely due to physical proximity to the worst single deletion or a hub. Ultimately, considering an “epicenter” region as a given cortical region that is larger than a single parcellation would meaningfully allow us to predict the likely most important region for a given connectotype. It is worth noting that one should not over-emphasize small differences in which the first or second worst parcellation was given because these differences could be due to small nuanced differences calculated by our algorithm or in fiber tracking.

The data presented in Supplementary Table 2 demonstrated that for every lobe in the right and left hemisphere, specific epicenters existed such that even if the worst single parcellation was not at the center of an epicenter, this parcellation would still show up in the progression earlier than expected. This may suggest that it is disproportionately important. Interestingly, for every lobe, at least 2 or 3 epicenter parcellations could be identified as either a common worst single deletion or early arrival in the progression compared to physical distance; yet based on the worst *single* parcellation deletion, the epicenter for specific subjects differed. This suggested that each lobe in each subject could have specific differences in which a general part of the lobe was worse or better but that this was not infinitely complex. Instead, it followed general patterns indicating wherethe epicenter of worst injury probability lies. We suggest that this raises the possibility of specific connectotypes which could be beneficial in patient-specific neurosurgicalplanning. By identifying a person’s connectotype, one could reasonably predict which parcellation is most likely the worst parcellation to remove and thus determine the general region to avoid even if another adjacent parcellation came up as the worst in our deletion list.

**Figure 2:**
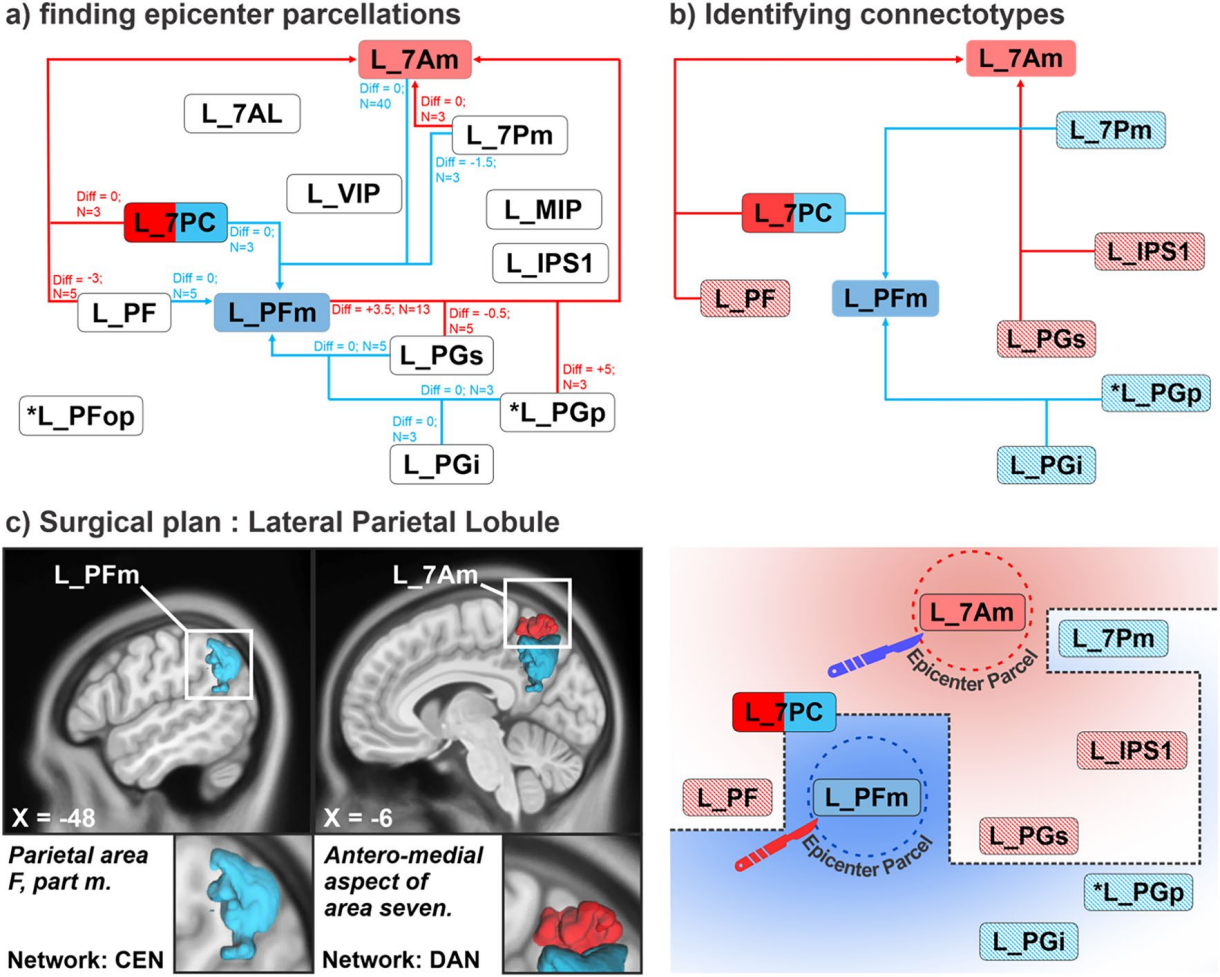
Finding surgically actionable differences in inter-individual “Connectotypes”. (**a**) A schematic diagram showing variation in the relationship between individual parcellations and the worst deletion area according to the largest drop in GE scores or early arrival in the deletion progression compared to physical distance. Areas 7Am and PFm were the most common worse areas between the majority of individuals. A subset of individuals (*) also demonstrated areas PFop or PGp as the worst deletion areas. However, in this same subset of individuals, area PFm would still show up in the deletion progression sequence earlier than expected compared to physical distance alone, suggesting that it is disproportionately more important than other regions even if it is not the single worst deletion area, which could be due to small differences in fiber tracking. (**b**) The worst regions (areas 7Am and PFm) form the core of two unique epicenters which suggested that individuals could differ between which region of a lobe was the worst and should be avoided in surgery. (**c**) These epicenter parcellations and their relationship with neighboring parcellations can be generally grouped into unique “connectotypes” which provide surgically actionable anatomic information that reflects fundamental differences between two individuals which led to differences in the progression of worst deletions at each extent of deletion. For instance, individuals with area PFm at the core of an epicenter have a laterally dominant connectotype and this information could inform a preference for a surgical approach that is positioned more supero-medially (blue scalpel). Red: the most common epicenter in the respective lobe. Blue: the second most common epicenter in the respective lobe. Two colored rectangles: cortical areas with shared epicenters. Asterisk: individual variability with different epicenters as shown. Diff: difference between the actual sequence and minimum path length between two connected areas. Minimal path length is referring to the minimum distance between two connected areas. Actual sequence is referring to the number of sequences for the connected epicenter to be shown in the deletion list. N: the number of individuals who have the respective worst area in deletion. Blue lines represent one unique connectotype compared to a different connectotype with red lines.

An example of this can be shown in Fig. 2 for the lateral parietal lobe in the left hemisphere. Individuals demonstrated two unique connectotypes in the left hemisphere which had two unique epicenters: areas L_PFm in the supramarginal gyrus and L_7Am in the superior parietal lobule were found to be the worst deletions. These parcellations were the worst in 43 and 27 individuals, respectively, and were the core of two unique epicenters. However, in a small subset of individuals, areas L_PFop or L_PGp were found as the worst areas and possible core epicenter parcellations compared to L_7AM in the lateral parietal lobe since the actual sequence for L_7AM to show up in the list was later than the expected distance. Ultimately, when these parcellations (L_PFop and L_PGp) were found as the single worst areas in a small subset of individuals, area L_PFm showed up immediately second. Therefore, while these small differences in rank for the worst deletion may be due to small, nuanced differences calculated by our algorithm or for specific fibers measured with fiber tracking, these individuals all likely belonged to the same laterally dominant connectotype with a significant epicenter focused around area L_PFm compared to medially dominant individuals with a connectotype that included an epicenter region around area L_7Am. This grouping allows us to understand the relationship between the worst parcellations in a clinically actionable way which can be utilized in surgery to avoid a single area rather than a single parcellation.

Results for each individual lobe detail the specific connectotypes and epicenter regions found for each of the 8 cortical regions per cerebral hemisphere in Supplementary Figs. 1–8.

## Discussion

A poor understanding of the mechanism of interindividual variability of cognitive outcomes for patients undergoing brain surgery has real world consequences that affect quality of life and likely reduces the effectiveness of oncologic therapy. While gross anatomic variation is one possible explanation as the reason for why the same intervention can often have substantially different consequences in different patients, the current study demonstrates that among individuals there exists different patterns of the most detrimental surgical transgression in a cerebral lobe. More specifically, we found that these patterns focus around epicenters within specific “connectotypes” that followed predictable, distinct, and spatially separate patterns and provide significant clinical importance for neurosurgeons in an actionable way.

Brain surgery involves making difficult decisions about where to cut and where to avoid. While intra-axial brain surgery as presently practiced is not precise to the level of small cortical regions on the scale of individual parcellations, knowing which general part of a lobe is worse than other parts is critical to planning trajectories to deep targets and assessing risk and benefit of a proposed procedure^45^. One appealing aspect of the “connectotype” concept is that by determining an epicenter of a lobe for a patient, a specific angle of approach can be prioritized or disfavored at a surgically actionable scale. For instance, access to deep lesions in the atria of the lateral ventricle is commonly achieved through a variety of approaches which often transgress the parietal lobe. Specifically, preferred approaches commonly use a trajectory through the superior parietal lobe, such as the transcortical “SPL approach”^46^, since the SPL has been traditionally considered as a functionally “silent” region especially in comparison to the adjacent “eloquent” inferior parietal lobe^47^. However, some patients may face disproportionately worse outcomes when operating in this area despite the same surgical approach^46^, and these differences while previously unpredictable may be partly explainable when considering a patient’s connectotype. For patients with a supero-medially dominant connectotype with an epicenter around area 7Am of the SPL in the left hemisphere (Fig. 2), a different surgical corridor to the target could be considered to optimize post-operative morbidity rather than directly through the brain parenchyma in a transcortical approach^48^, such as a right-sided interhemispheric approach through the corpus callosum^49,50^ or even just a more oblique and less paramedian approach^46^. These subtle differences in the surgical approach to reach similar underlying regions can drive drastic differences in neuropsychological sequelaebut may be addressed preoperatively with consideration of general differences in patient connectotypes.

In this study, brain regions were divided based on the HCP atlas as outlined by Glasser et al.^30^ because this parcellation scheme has been studied extensively by our group and others as well^34,51–53^. Achieving a comprehensive understanding of the brain becomes challenging when diverse naming conventions or node creation methods are employed, so by choosing one parcellation scheme to study in our group, we can hope to grow a body of literature around it. Additionally, the connectivity of healthy subjects rather than individuals with infiltrating tumors was selected for this study because our goal was to first understand normal variation in healthy individuals. It is true that gliomas and other malignant tumors may lead to structural and functional connectivity that differs from that of normal, healthy subjects. However, given the high degree of variability in the data from patients with tumors, it would be extremely difficult to interpret abnormal results if we cannot yet interpret normal variation. This study could therefore serve as a foundation for future research using connectivity of patients with malignant tumors. The theoretical brain surgery model we studied applied optimal percolation theory on structural networks to investigate a location-specific pattern of injury on a cortical network’s efficiency predicted in the operative period^29,43,54^. Structural networks place important constraints on a network’s functional connectivity and overall efficiency^11,55^ and therefore have especially important implications in neurosurgery^56,57^. While structural networks are known to be generally resistant to a degree of insult^43^, optimal percolation theory allows us to plausibly identify critical nodes which most strongly maintain a network’s efficiency as measured by GE^29^. Critical nodes could potentially demonstrate higher metabolic activity to sustain its increased connectivity to a network^58^. Consequently, they may be particularly prone to adverse effects associated with neurodegenerative processes^59^, localization of gliomas^60^, and functional damage after surgical insult^61^. Indeed, our results partially agree with previous work in that cortical networks are especially vulnerable to targeted perturbations at critical nodes which can often be defined by their centrality and therefore are useful to consider for neurosurgery. However, it is important to note that there were several additional parcellations which were not found to be highly central hubs even when we included neighboring hub parcellations and yet were highly detrimental to GE after deletion (2–17%; Table 3). Considering such discrepancies, while not necessarily drastic by numeric measures, is clinically imperative in resective brain surgery on an individual basis given the extremely limited amount of error which is allowed in surgical decision making before inducing functional consequences. The concept of epicenters which defined specific patient connectotypes offers one possible solution to address this concern in a clinically actionable way.

In conclusion, theoretical brain surgery utilizing graph theory metrics like GE provides a more insightful approach to neurosurgery by informing surgeons how to understand and predict the consequences of specific surgical decisions. Neurosurgeons could carry out disproportionate damage by making very small surgical decisions that generally do not affect the surgery but also do not take into account differences in brain architecture based on individual variability. There is a location-specific pattern to these injuries in which there are common epicenters that include the worst regions which constitute the greatest consequences on a brain network’s efficiency when damaged. However, these areas are not all equal between every individual and therefore must be considered if we are to optimize patient cognitive morbidity following neurosurgery. Considering the progress in neuro-navigation and parcellation targeting, further research is warranted to confirm the findings of this current study through lesion or transection studies conducted in non-human primates. It is also worth mentioning that other graph measures alone or in combination with GE may also be useful in quantifying postoperative deficits and improve the creation of personalized neurosurgical plans, but additional research is necessary to validate these approaches^62,63^. Future work may also attempt to correlate clinical outcomes with percolated structural networks in a prospective manner following localized brain surgery^64^. Linking ideas from the current study with such investigations provides a critical goal moving forward to continue to maximize the patient's onco-functional balance.

### Supplementary Information

Supplementary Information.

## Data Availability

All relevant data has been supplied in the current manuscript. Should the reader request additional data, the corresponding author may be contacted for further information (Michael Sughrue, sughruevs@gmail.com). Healthy participant data was obtained from randomly selected participants from the publicly available OpenNeuro (https://openneuro.org) dataset. Participant IDs were provided in Supplementary Table 1.

## Abbreviations

CNN: Convolutional neural net
DTI: Diffusion tensor image
HCP: Human connectome project
GE: Global efficiency
MRI: Magnetic resonance images
PR: PageRank
ROI: Region of interests
WHO: World Health Organization
