# Supplementary material for: Discernible interindividual patterns of global efficiency decline during theoretical brain surgery
Source: Sci Rep. 2024 Jun 25;14:14573. doi: 10.1038/s41598-024-64845-4 (PMC11196730; doi:10.1038/s41598-024-64845-4)
Supplement: Supplementary file 1 — Supplementary Information. [file 41598_2024_64845_MOESM1_ESM.docx]

**Supplementary Material: DISCERNIBLE INTERINDIVIDUAL PATTERNS OF GLOBAL EFFICIENCY DECLINE DURING THEORETICAL BRAIN SURGERY**

Lin Yueh-Hsin^1*^, Nicholas B. Dadario^2*^, Si Jie Tang^3^, Lewis Crawford^4^, Onur Tanglay^4^ , Hsu-Kang Dow^5^, Isabella Young^4^, Syed Ali Ahsan^1^, Stephane Doyen^4^, Michael E. Sughrue^1,4^

**Author affiliation:**

^1^Centre for Minimally Invasive Neurosurgery, Prince of Wales Private Hospital, Suite 19, Level 7 Prince of Wales Private Hospital, Randwick, Sydney, NSW 2031, Australia.

^2^Robert Wood Johnson Medical School, Rutgers University, 125 Paterson St, New Brunswick, NJ 08901, United States of America

^3^Omniscient Neurotechnology, Level 10/580 George Street, Sydney, NSW, 2000, Australia

^4^ School of Computer Science and Engineering, University of New South Wales, Building K17 UNSW Sydney, NSW 2052

Correspondence to: Michael E. Sughrue

Centre for Minimally Invasive Neurosurgery

Prince of Wales Private Hospital

Suite 3, Level 7 Barker St

Randwick NSW 2031

Tel: (02) 9650 4940

Email: sughruevs@gmail.com

**Supplementary Methods**

| **Total number of subjects** | **Participant ID** | **Age** | **Gender** |
| --- | --- | --- | --- |
| 1 | sub-10171 | 24 | M |
| 2 | sub-10189 | 49 | M |
| 3 | sub-10206 | 21 | M |
| 4 | sub-10217 | 33 | F |
| 5 | sub-10227 | 31 | F |
| 6 | sub-10228 | 40 | F |
| 7 | sub-10235 | 22 | M |
| 8 | sub-10290 | 48 | M |
| 9 | sub-10304 | 23 | F |
| 10 | sub-10321 | 34 | M |
| 11 | sub-10325 | 29 | F |
| 12 | sub-10329 | 22 | M |
| 13 | sub-10339 | 45 | F |
| 14 | sub-10340 | 32 | M |
| 15 | sub-10345 | 25 | M |
| 16 | sub-10347 | 25 | F |
| 17 | sub-10356 | 43 | F |
| 18 | sub-10361 | 42 | F |
| 19 | sub-10365 | 24 | F |
| 20 | sub-10376 | 42 | F |
| 21 | sub-10377 | 49 | M |
| 22 | sub-10388 | 50 | F |
| 23 | sub-10429 | 36 | F |
| 24 | sub-10438 | 25 | M |
| 25 | sub-10440 | 26 | F |
| 26 | sub-10448 | 37 | M |
| 27 | sub-10455 | 25 | M |
| 28 | sub-10460 | 40 | M |
| 29 | sub-10471 | 30 | F |
| 30 | sub-10478 | 21 | F |
| 31 | sub-10487 | 31 | M |
| 32 | sub-10492 | 22 | F |
| 33 | sub-10506 | 25 | M |
| 34 | sub-10517 | 21 | F |
| 35 | sub-10523 | 24 | M |
| 36 | sub-10524 | 46 | F |
| 37 | sub-10525 | 24 | M |
| 38 | sub-10527 | 24 | M |
| 39 | sub-10557 | 26 | M |
| 40 | sub-10565 | 25 | F |
| 41 | sub-10570 | 32 | F |
| 42 | sub-10575 | 23 | M |
| 43 | sub-10624 | 28 | M |
| 44 | sub-10629 | 31 | M |
| 45 | sub-10631 | 21 | F |
| 46 | sub-10638 | 25 | F |
| 47 | sub-10674 | 42 | F |
| 48 | sub-10678 | 24 | F |
| 49 | sub-10680 | 22 | F |
| 50 | sub-10686 | 26 | F |
| 51 | sub-10692 | 28 | F |
| 52 | sub-10696 | 25 | F |
| 53 | sub-10697 | 39 | M |
| 54 | sub-10704 | 32 | M |
| 55 | sub-10707 | 28 | M |
| 56 | sub-10708 | 25 | M |
| 57 | sub-10719 | 23 | F |
| 58 | sub-10724 | 22 | F |
| 59 | sub-10746 | 23 | M |
| 60 | sub-10762 | 41 | F |
| 61 | sub-10779 | 38 | M |
| 62 | sub-10785 | 36 | F |
| 63 | sub-10788 | 35 | F |
| 64 | sub-10844 | 26 | M |
| 65 | sub-10855 | 47 | M |
| 66 | sub-10871 | 41 | M |
| 67 | sub-10877 | 23 | M |
| 68 | sub-10882 | 23 | M |
| 69 | sub-10891 | 21 | F |
| 70 | sub-10893 | 25 | F |
| 71 | sub-10912 | 44 | F |
| 72 | sub-10934 | 37 | F |
| 73 | sub-10940 | 25 | M |
| 74 | sub-10948 | 22 | F |
| 75 | sub-10949 | 27 | M |
| 76 | sub-10958 | 30 | F |
| 77 | sub-10963 | 45 | F |
| 78 | sub-10968 | 24 | M |
| 79 | sub-10975 | 32 | M |
| 80 | sub-10977 | 47 | M |

**Supplementary Table 1.** Participant ID and characteristics

**Supplementary Results**

Below we detail the specific connectotypes and epicenter regions found for each of the 8 cortical regions per cerebral hemisphere analyzed. Specifically, these connectotypes are visualized in figures S1-8. The deletion combinations examined are shown in tables S2-9. Furthermore, the path length and sequence relationships between individual parcellations and the epicenter for each region are seen in extended table 10.

**Frontal lobe**

# Three different connectotypes were observed in the frontal lobe consisting of three distinct epicenters in both the left and right hemisphere. Left 45, l_10d and 8Av were the main epicenter parcellations in the left frontal lobe (1a), which had 29, 15 and 14 individuals that share these common patterns, respectively. In a small group of individuals, L_8BL could be a more significant area than L_45 and L_8Av as the worst area in the left frontal lobe, as the actual sequence for L_45 and L_8Av to show up in the list were later than the expected distance. In addition, L_47l and L_10pp could be a more significant area than L_10d as it showed up in the list later than the expected distance. However, grouping the parcellations into various epicenters had shown that L_9a and L_10pp belonged to same L_45 epicenter parcellation connectotype, while L_8BL, L_a47r and L_47l belonged to L_8Av epicenter parcellation connectotype. For L_8C and L_44, both belonged to both L_45 and L_8Av epicenter parcellation connectotype.

#
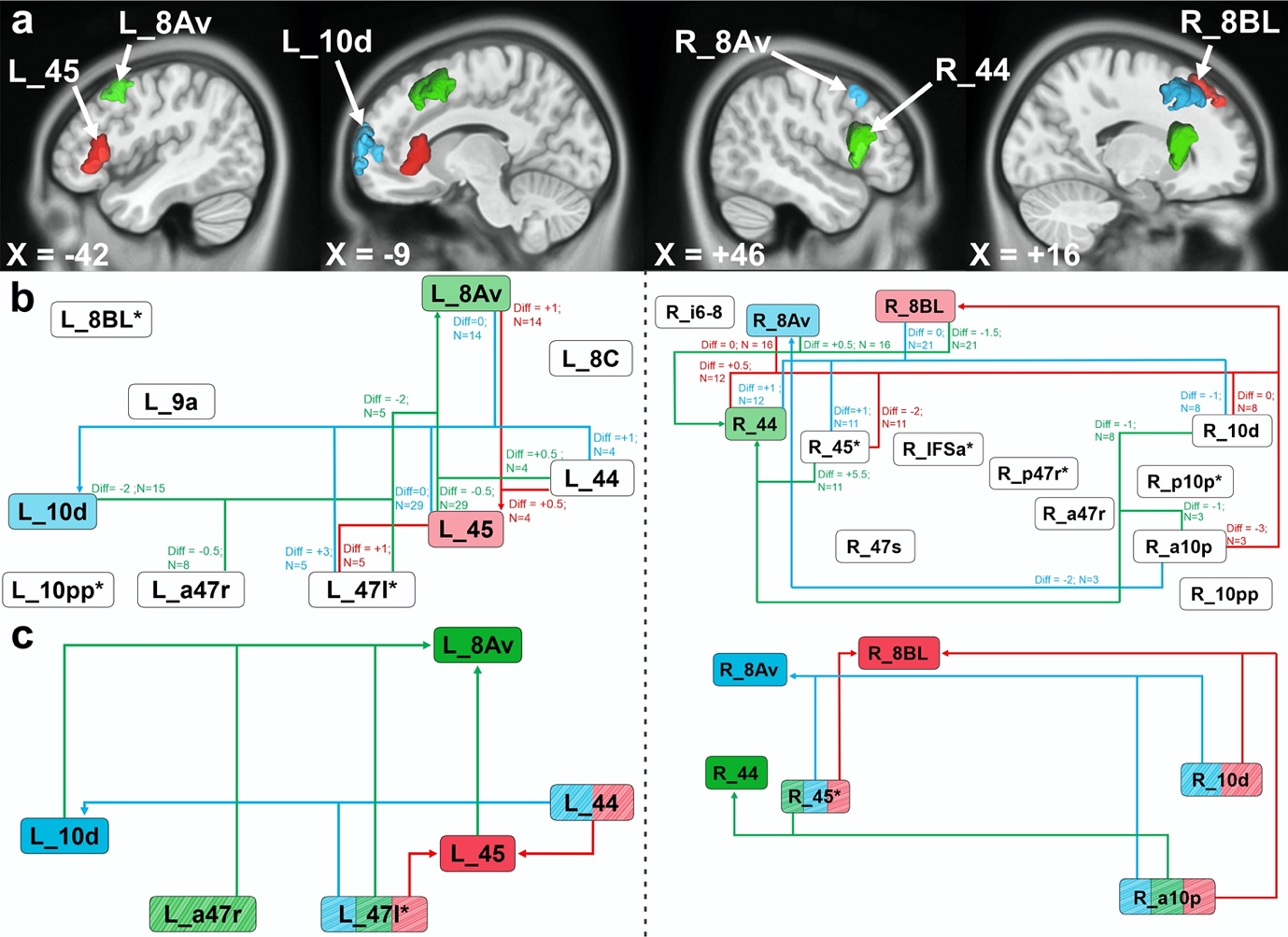
In the right hemisphere (1b), right 8BL, 8Av and 44 were the main epicenter parcellations, with 21, 16 and 12 individuals sharing these common patterns, respectively. In a small subset of individuals, R_45 and R_IFSa were found as the worst areas compared to R_8Av and R_44. Furthermore, although the number of individuals with this characteristic was less significant, the worst areas such as R_p47r could present a more significant epicenter parcellation than areas R_8Av and R_44 in the right frontal lobe, as the actual sequence for R_8Av and R_44 to show up in the list were later than the expected distance. Also, for individuals with R_p10p as the worst area, it was observed that R_8BL was a less significant epicenter parcellation. However, after analyzing the relationships between the worst deletion regions, R_45, R_IFSa, R_p10p, R_a10p and R_10pp belonged to the same connectotype with an R_8BL epicenter parcellation, R_47s belonged to the R_8Av epicenter parcellation connectotype, and R_i6-8 belonged to the R_44 epicenter parcellation connectotype. R_p47r and R_10d belonged to both R_8Av and R_44 centered connectotypes, while R_a47r belonged to the R_8BL and R_8Av epicenter parcellation connectotypes, respectively.

**SI Figure 1.** Sagittal sections show the worst deletion areas which form the core of an epicenter for a specific connectotype, known as the epicenter parcellations, in frontal lobe of the left (a) and right (b) hemispheres. A schematic diagram shows variations among individuals with the worst cortical region in the left (c) and right (d) frontal lobes. A schematic diagram grouping the worst cortical regions into different epicenters in the left (e) and right (f) frontal lobes. Red: the most common epicenter in the respective lobe. Blue: the second most common epicenter in the respective lobe. Green: the third most common epicenter in the respective lobe. Two colored rectangles: cortical areas with shared epicenters. Asterisk: individual variability with different epicenters as shown. Diff: difference between the actual sequence and minimum path length between two connected areas. Minimal path length is referring as the minimum distance between two connected areas. Actual sequence is referring as the number of sequences for the connected epicenter to be shown in the deletion list. N: the number of individuals who has the respective worst area in deletion.

| **2. Frontal lobe** | | | |
| --- | --- | --- | --- |
| **Size of deletion** | | **Size of deletion** | |
| **1** | 29/80 L_45 | 1 | 21/80 R_8BL |
| **2** | 33/80 L_44, **L_45** | 2 | 21/80 R_44, R_45 |
| **3** | 20/80 L_a47r, **L_45**, L_47L | 3 | 21/80 R_8AV, R_8AD, **R_8BL** |
| **4** | 22/80 **L_a47r, L_45, L_44, L_47L** | 4 | 12/80 **R_8AV, R_8AD, R_8BL**, R_i6-8 |
| **5** | 8/80 **L_a47r, L_45, L_44,** L_a9-46v, **L_47L** | 5 | 6/80 **R_8AV, R_i6-8, R_8AD, R_8BL**, R_s6-8 |
| **5** | 8/80 L_8AV, L_IFSp, L_8C, **L_45, L_44** | | 6/80 **R_8AV**, R_46, R_IFSa, **R_45**, **R_44** |
| **6** | 9/80 **L_a47r**, L_10pp, L_10d, **L_45**, L_p47r, L_a10p | 6 | 5/80 R_a47r, R_10pp, R_10d, **R_45**, R_p47r, R_a10p |
| **7** | 6/80 **L_a47r, L_10pp, L_10d, L_45, L_p47r, L_a10p, L_47L** | 7 | 5/80 **R_8AV, R_i6-8, R_8AD, R_10d**, R_9p, **R_8BL**, R_9a |
| **8** | 9/80 **L_a47r, L_10pp, L_10d**, L_p10p, **L_45, L_p47r, L_a10p**, **L_47L** | 8 | 6/80 **R_a47r, R_10pp, R_10d**, R_p10p, **R_IFSa, R_45, R_p47r, R_a10p** |
| **9** | 9/80 **L_8AV, L_a47r,** L_46, **L_8C,** L_IFSa, **L_45, L_p47r**, L_p9-46v, **L_47L** | 9 | 8/80 **R_8AV**, R_46, **R_i6-8**, R_IFSp, **R_8AD, R_8BL**, R_8C, R_s6-8, **R_44** |
| **10** | 13/80 **L_8AV, L_46, L_IFSp, L_8C**, L_IFJa, **L_45, L_44, L_p47r, L_p9-46v, L_47L** | 10 | 19/80 **R_8AV, R_46, R_i6-8, R_IFSp**, **R_8AD, R_8BL, R_8C, R_s6-8,** R_IFJa, **R_44** |

**Supplementary Table S2.** List of the worst parcellation combinations deleted among 80 individuals in the frontal lobe. Parcellations that are bolded mean the occurrence of the worst deletion in previous combination. Progression of deleted combination has shown to be an accumulation of previous worst areas in percolation.

**Sensorimotor cortex**

Individuals demonstrated two unique connectotypes in the left hemisphere (2a) and three different connectotypes in the right hemisphere (2b). Left SFL and 6ma were the main epicenter parcellations in the left sensorimotor cortex, with 48 and 12 individuals sharing these common patterns, respectively. In a small subset of individuals, areas L_6d and L_55b were found to be more significant areas than L_SFL and L_6ma in the left sensorimotor cortex, as the actual sequence for L_SFL and L_6ma to show up in the list were later than the expected distance. Therefore, for individuals with L_6d and L_55b as the worst areas, their epicenter progression starts from the lateral regions instead of the medial surface. To understand the relationship between the worst parcellations in a clinically actionable way, grouping the parcellations into various epicenters had shown unique connectotypes in which areas L_6mp and L_4 belonged to L_SFL epicenter parcellation connectotype, while L_55b and L_5L belonged to L_6ma epicenter parcellation connectotype. L_1 and L_6d belonged to both L_SFL and L_6ma epicenter parcellation connectotypes, respectively.

# In the right sensorimotor cortex, the right SFL, 4 and 6ma were the main epicenter parcellations, with 30, 16 and 13 individuals sharing these common patterns, respectively. A small amount of individuals demonstrated R_5L as possibly the worst area. For individuals with R_5L as the worst area, R_5L was more closely related to the R_4 epicenter than the R_ SFL. These results demonstrate that subjects have their epicenters located at the posterior region of the somatosensory area. Grouping the worst parcellations into different connectotypes focused around unique epicenters, in the right hemisphere R_5L belonged to R_SFL epicenter parcellation connectotype, while R_6d and R_6v belonged to R_4 epicenter parcellation connectotype. R_1 belonged to both R_SFL and R_6ma epicenter parcellation connectotypes in the superior and anterior surface of the frontal lobe.


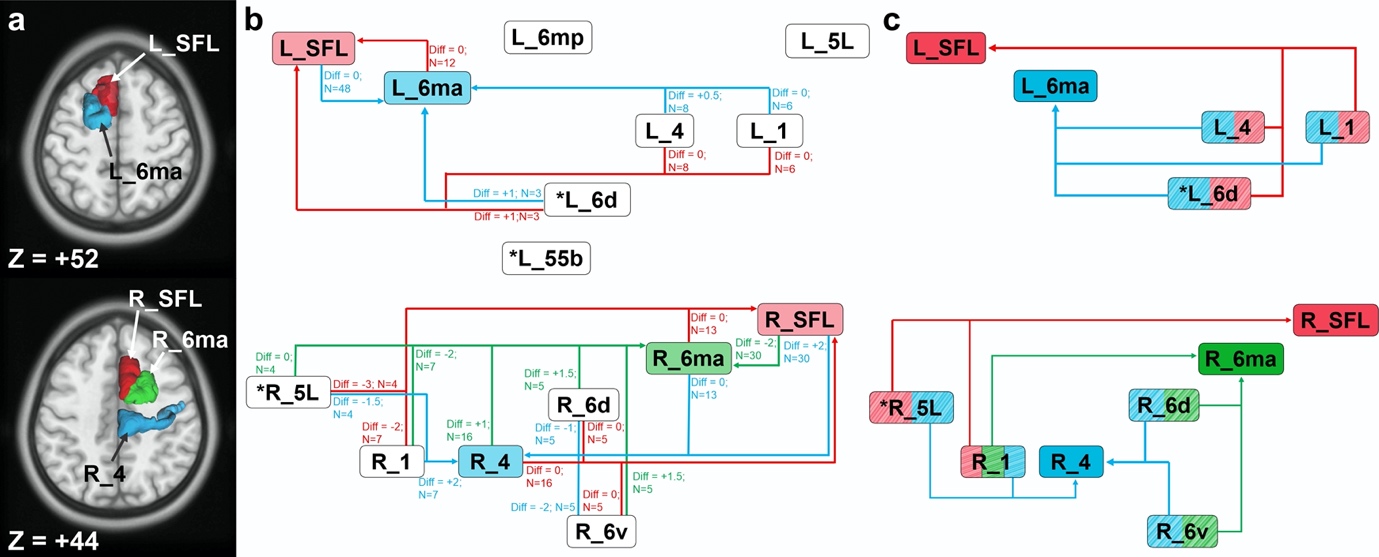


**SI Figure 2.** Axial sections show the worst deletion areas which form the core of an epicenter for a specific connectotype, known as the epicenter parcellations, in the sensorimotor cortex of the left (a) and right (b) hemispheres. A schematic diagram showing the relationship between epicenter parcellations and neighboring parcellations is shown for the left (c) and right (d) sensorimotor cortices. A schematic diagram of varying connectotypes consisting of specific epicenters and neighboring parcellations in the left (e) and right (f) sensorimotor cortices. Red: the most common epicenter in the respective lobe. Blue: the second most common epicenter in the respective lobe. Green: the third most common epicenter in the respective lobe. Two coloured rectangles: cortical areas with shared epicenters. Asterisk: individual variability with different epicenters as shown. Diff: difference between the actual sequence and minimum path length between two connected areas. Minimal path length is referring to the minimum distance between two connected areas. Actual sequence is referring to the number of sequences for the connected epicenter to be shown in the deletion list. N: the number of individuals who have the respective worst area in deletion.

| **3. Sensorimotor cortex** | | | |
| --- | --- | --- | --- |
| Size of deletion | | Size of deletion | |
| 1 | 48/80 L_SFL | 1 | 30/80 R_SFL |
| 2 | 53/80 L_6ma, **L_SFL** | 2 | 38/80 R_6ma, **R_SFL** |
| 3 | 47/80 L_6mp, **L_6ma, L_SFL** | 3 | 31/80 R_6mp, **R_6ma, R_SFL** |
| 4 | 48/80 **L_6mp,** L_4, **L_6ma, L_SFL** | 4 | 38/80 **R_6mp**, R_4, **R_6ma, R_SFL** |
| 5 | 59/80 **L_4, L_6ma**, L_6d, **L_6mp, L_SFL** | 5 | 36/80 **R_4, R_6ma**, R_6d, **R_6mp, R_SFL** |
| 6 | 28/80 **L_4, L_6ma, L_6d**, L_55b, **L_6mp, L_SFL** | 6 | 37/80 R_6v, **R_4, R_6ma, R_6d, R_6mp, R_SFL** |
| 7 | 46/80 L_6v, **L_4, L_6ma, L_6d, L_55b, L_6mp, L_SFL** | 7 | 50/80 **R_6v, R_4, R_6ma, R_6d**, R_55b, **R_6mp, R_SFL** |
| 8 | 46/80 **L_6v, L_4, L_6ma**, L_6a, **L_6d, L_55b, L_6mp, L_SFL** | 8 | 51/80 **R_6v, R_4, R_6ma**, R_6a, **R_6d, R_55b, R_6mp, R_SFL** |
| 9 | 70/80 L_PEF, **L_6v, L_4, L_6ma, L_6a, L_6d, L_55b, L_6mp, L_SFL** | 9 | 54/80 R_PEF, **R_6v, R_4, R_6ma, R_6a, R_6d, R_55b, R_6mp, R_SFL** |
| 10 | 62/80 **L_PEF, L_6v, L_4, L_6ma, L_6d, L_6a,** L_3a, L_5m, **L_55b, L_6mp** | 10 | 53/80 **R_PEF, R_6v, R_4, R_6ma, R_6d, R_6a**, R_3a, R_5m, **R_55b, R_6mp** |

**Supplementary Table S3.** List of the worst parcellation combinations deleted among 80 individuals in the sensorimotor cortex. Parcellations that are bolded mean the occurrence of the worst deletion in previous combination. Progression of deleted combination has shown to be an accumulation of previous worst areas in percolation.

**Medial frontal lobe**

Individuals demonstrated two unique connectotyps in the left hemisphere (3a) and three different connecotypes in the right hemisphere (3b) for the medial frontal lobe. In the left medial frontal lobe, L_9m and L_SCEF were the main epicenter parcellations, which had 31 and 24 individuals sharing these common patterns, respectively. However, despite the fact that the number of individuals with this characteristic is less significant, L_pOFC could be a worse area than L_9m in the medial frontal lobe as the actual sequence for L_9m to show up in the list is later than the expected distance. After we further analysed the grouping of parcellations, L_pOFC belonged to L_SCEF epicenter parcellation connectotype in the posterior surface of the medial frontal lobe, while L_10v and L_24dd belonged to L_9m epicenter parcellation connectotype in the anterior surface of the medial frontal lobe. L_a24 belonged to both L_9m and L_SCEF epicenter parcellation connectotype.

In the right medial frontal lobe, R_9m, R_SCEF and R_10v were the top worst areas in the right medial lobe. Individuals with R_24dd and R_pOFC as the worst deletions have been shown to have different epicenters. For individuals with R_24dd found as the worst area, R_24dd could more closely be related to R_SCEF and R_9m epicenter connectotype than the R_10v epicenter connectotype. Therefore, these individuals have epicenters that are located at the posterior region of the medial frontal lobe. Individuals with R_pOFC as the epicenter parcellation were more closely related to R_SCEF and R_9m epicenter parcellation connectotypes than the R_10v connectotype. Therefore, it was estimated that the epicenters for these individuals emerge from two distinct points: the anterior frontal and the posterior medial frontal regions. R_11l and R_13l have shown to be more closely related to epicenter R_10v. Hence, individuals with R_11l and R_13l as the worst area would have their epicenter progression originate from the frontal pole. After grouping the worst cortical regions into different connectotypes around unique epicenters, R_pOFC, R_13l and R_8BM belonged to R_9am epicenter parcellation connectotype, while R_24dd was shown to belonged to R_10v epicenter parcellation connectotype. For R_11l, it belonged to both R_9m and R_SCEF epicenter parcellation connectotypes.

**
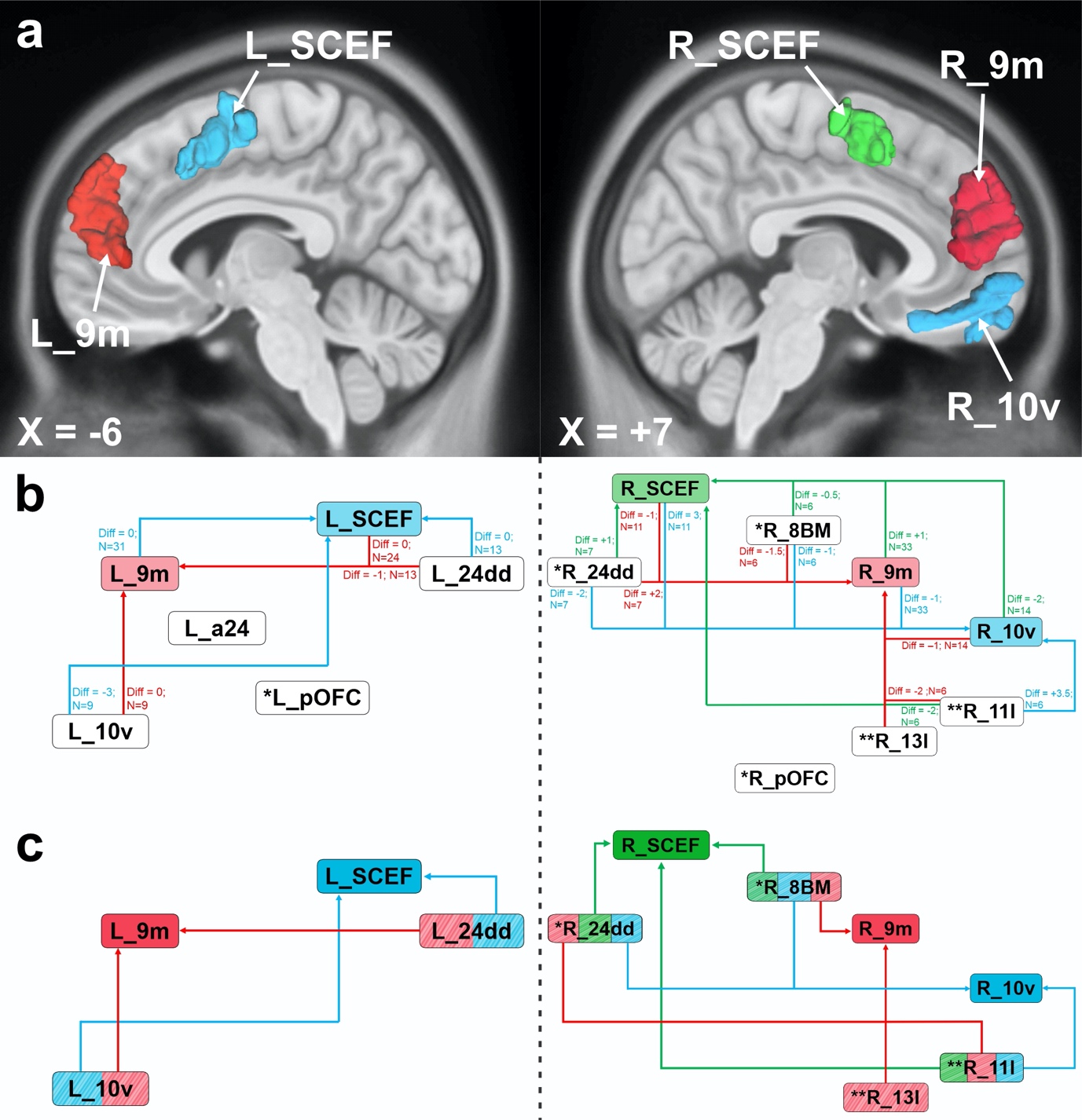
**

**SI Figure 3.** Sagittal sections show the worst deletion areas which form the core of an epicenter for a specific connectotype, known as the epicenter parcellations, in the medial frontal lobe of the left (a) and right (b) hemispheres. A schematic diagram showing the relationship between epicenter parcellations and neighboring parcellations is shown for the left (c) and right (d) medial frontal lobes. A schematic diagram of varying connectotypes consisting of specific epicenters and neighboring parcellations in the left (e) and right (f) medial frontal lobes.

Red: the most common epicenter in the respective lobe. Blue: the second most common epicenter in the respective lobe. Green: the third most common epicenter in the respective lobe. Two coloured rectangles: cortical areas with shared epicenters. Asterisk: individual variability with different epicenters as shown. Diff: difference between the actual sequence and minimum path length between two connected areas. Minimal path length is referring to the minimum distance between two connected areas. Actual sequence is referring to the number of sequences for the connected epicenter to be shown in the deletion list. N: the number of individuals who have the respective worst area in deletion

| **4. Medial frontal lobe** | | | |
| --- | --- | --- | --- |
| **Size of deletion** | | **Size of deletion** | |
| 1 | 31/80 L_9m | 1 | 33/80 R_9m |
| 2 | 44/80 L_24dd, L_SCEF | 2 | 37/80 R_8BM, **R_9m** |
| 3 | 41/80 **L_9m, L_SCEF**, L_8BM | 3 | 47/80 **R_9m**, R_SCEF, **R_8BM** |
| 4 | 66/80 **L_9m, L_24dd, L_SCEF, L_8BM** | 4 | 57/80 **R_9m**, R_24dd, **R_SCEF, R_8BM** |
| 5 | 20/80 **L_8BM, L_24dd, L_SCEF, L_9m**, L_a24 | 5 | 16/80 **R_8BM, R_24dd**, R_p32pr, **R_SCEF, R_9m** |
| 6 | 10/80 **L_8BM**, L_p24, **L_24dd, L_SCEF, L_9m, L_a24** | 6 | 8/80 **R_8BM, R_24dd**, R_d32, **R_SCEF, R_9m**, R_a24 |
| 7 | 9/80 **L_8BM, L_p24, L_24dd**, L_a32pr, **L_SCEF, L_9m, L_a24** | 7 | 6/80 **R_8BM, R_24dd, R_d32, R_SCEF, R_9m**, R_24dv, **R_a24** |
| 8 | 14/80 **L_8BM, L_p24, L_24dd, L_a32pr**, L_d32, **L_SCEF, L_9m, L_a24** |  | 6/80 **R_8BM, R_24dd, R_d32, R_p32pr, R_SCEF, R_9m**, R_24dv |
| 9 | 21/80 **L_8BM, L_p24, L_24dd, L_a32pr, L_d32, L_SCEF, L_9m**, L_24dv, **L_a24** | 8 | 14/80 **R_8BM**, R_p24, **R_24dd, R_d32**, **R_p32pr, R_SCEF, R_9m, R_24dv** |
| 10 | 19/80 L_p24pr, L_a24pr, **L_8BM, L_a32pr, L_p24, L_d32,** L_p32pr, **L_SCEF, L_9m, L_24dv** | 9 | 22/80 **L_8BM**, L_p32, **L_p24, L_24dd, L_d32, L_SCEF, L_9m, L_24dv, L_a24** |
|  |  | 10 | 23/80 R_p24pr, R_a24pr, **R_8BM**, R_a32pr, **R_p24, R_d32, R_p32pr, R_SCEF, R_9m, R_24dv** |

**Supplementary Table S4.** List of the worst parcellation combinations deleted among 80 individuals in the medial frontal lobe. Parcellations that are bolded mean the occurrence of the worst deletion in previous combination. Progression of deleted combination has shown to be an accumulation of previous worst areas in percolation.

**Operculum**

Individuals demonstrated two unique connectotypes in the left hemisphere (4a) and three different connectotypes in the right hemisphere (4b) for the medial frontal lobe. The main epicenter parcellations in the left and right medial frontal lobe were observed in Figure 2d. In the left operculum, L_A4 and L_PoI2 were the core epicenters, with 54 and 9 individuals sharing these common patterns. L_AAIC and L_MI belonged to L_PoI2 epicenter, while L_43, L_PSL, L_FOP4 and L_A5 belonged to L_A4 epicenter. L_ OP4 belonged to both L_A4 and L_PoI2.

In the right operculum, R_ A4, R_43 and R_PoI2 were the top worst areas in the right insula and opercular cortex, with 41, 13 and 12 individuals sharing these common patterns. R_PSL, R_OP4, R_A5 and R_STGa belonged to R_A4 in the right hemisphere, while R_AAIC belonged to R_PoI2.


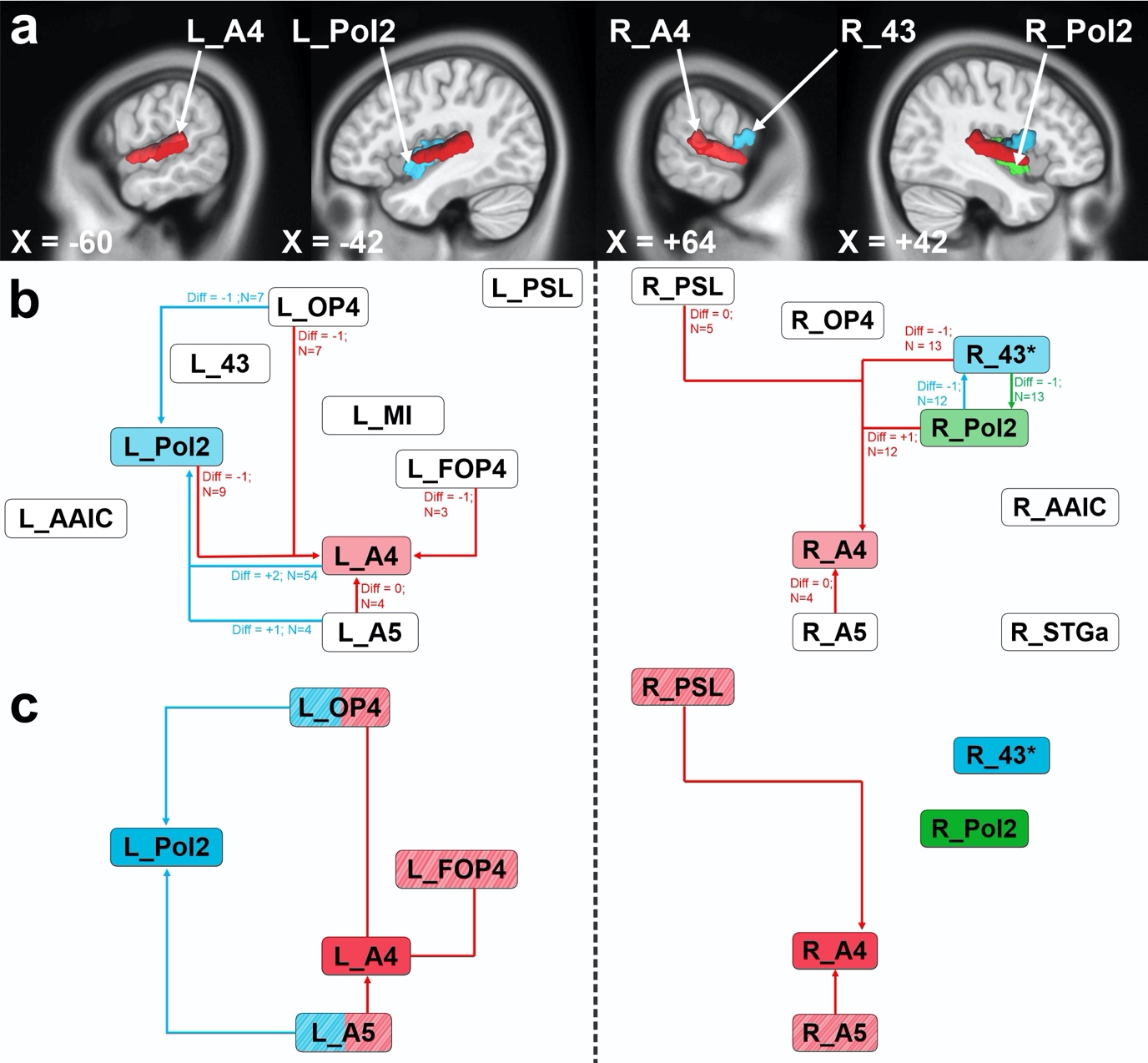


**SI Figure 4.** Sagittal sections show the worst deletion areas which form the core of an epicenter for a specific connectotype, known as the epicenter parcellations, in the insula and operculum of the left (a) and right (b) hemispheres. A schematic diagram showing the relationship between epicenter parcellations and neighboring parcellations is shown for the left (c) and right (d) insulo-opercular cortices. A schematic diagram of varying connectotypes consisting of specific epicenters and neighboring parcellations in the left (e) and right (f) insulo-opercular cortex. Red: the most common epicenter in the respective lobe. Blue: the second most common epicenter in the respective lobe. Green: the third most common epicenter in the respective lobe. Two colored rectangles: cortical areas with shared epicenters. Asterisk: individual variability with different epicenters as shown. Diff: difference between the actual sequence and minimum path length between two connected areas. Minimal path length is referring to the minimum distance between two connected areas. Actual sequence is referring to the number of sequences for the connected epicenter to be shown in the deletion list. N: the number of individuals who have the respective worst area in deletion.

| **5. Operculum** | | |  |
| --- | --- | --- | --- |
| **Size of deletion** | | **Size of deletion** | |
| 1 | 54/80 L_A4 | 1 | 41/80 R_A4 |
| 2 | 43/80 **L_A4**, L_A5 | 2 | 23/80 **R_A4,** R_A5 |
| 3 | 30/80 **L_A4, L_A5**, L_PSL | 3 | 24/80 **R_A4, R_A5**, R_PSL |
| 4 | 48/80 L_STV, **L_A4, L_A5, L_PSL** | 4 | 31/80 R_STV, **R_A4, R_A5, R_PSL** |
| 5 | 41/80 **L_A4, L_A5, L_PSL**, L_TA2, **L_STV** | 5 | 44/80 **R_A4, R_A5, R_PSL**, R_TA2, **R_STV** |
| 6 | 39/80 L_Pbelt, L_Lbelt, L_RI, **L_PSL**, L_Mbelt, L_PFcm | 6 | 42/80 R_Pbelt, R_Lbelt, R_RI, **R_PSL**, R_Mbelt, R_PFcm |

**Supplementary Table S5.** List of the worst parcellation combinations deleted among 80 individuals in the operculum. Parcellations that are bolded mean the occurrence of the worst deletion in previous combination. Progression of deleted combination has shown to be an accumulation of previous worst areas in percolation.

**Temporal lobe**

Individuals demonstrated two unique connectotypes in the left hemisphere (5a) and at least 2 different connectotypes in the right hemisphere (5b) for the temporal lobe. In the left temporal lobe, L_TGd and L_TE1p were the main epicenter parcellations, which had 43 and 27 individuals sharing these mutual patterns, respectively. Although only found in a subset of individuals, L_PHT and L_TE1m could be a more significant area than L_TGd in the temporal lobe as the actual sequence for L_TGd to show up in the list was later than the expected distance. Therefore, individuals with L_PHT and L_TE1m as the worst areas, their epicenter progression likely starts from the posterior and superior regions of the temporal lobe. Furthermore, most of the regions belonged to L_TE1p epicenter parcellation connectotype.

In the right temporal lobe, a connectotype with area R_ TGd as the main epicenter parcellation was found in the right temporal lobe, of which 52 individuals shared this common pattern of it being the worst area. Individuals with R_TE1p as the worst deletion have been shown to have different epicenters. Furthermore, despite their close anatomical relationship to R_TGd, individuals with R_TGv and R_PeEC appear to have separate epicenters. In the right temporal lobe, despite the dominance of R_TGd, areas including R_TE1p, R_TGv and R_PeEc were observed to have a separate epicenter. However, there were limited individuals who shared this characteristic, and further interpretation was challenging.


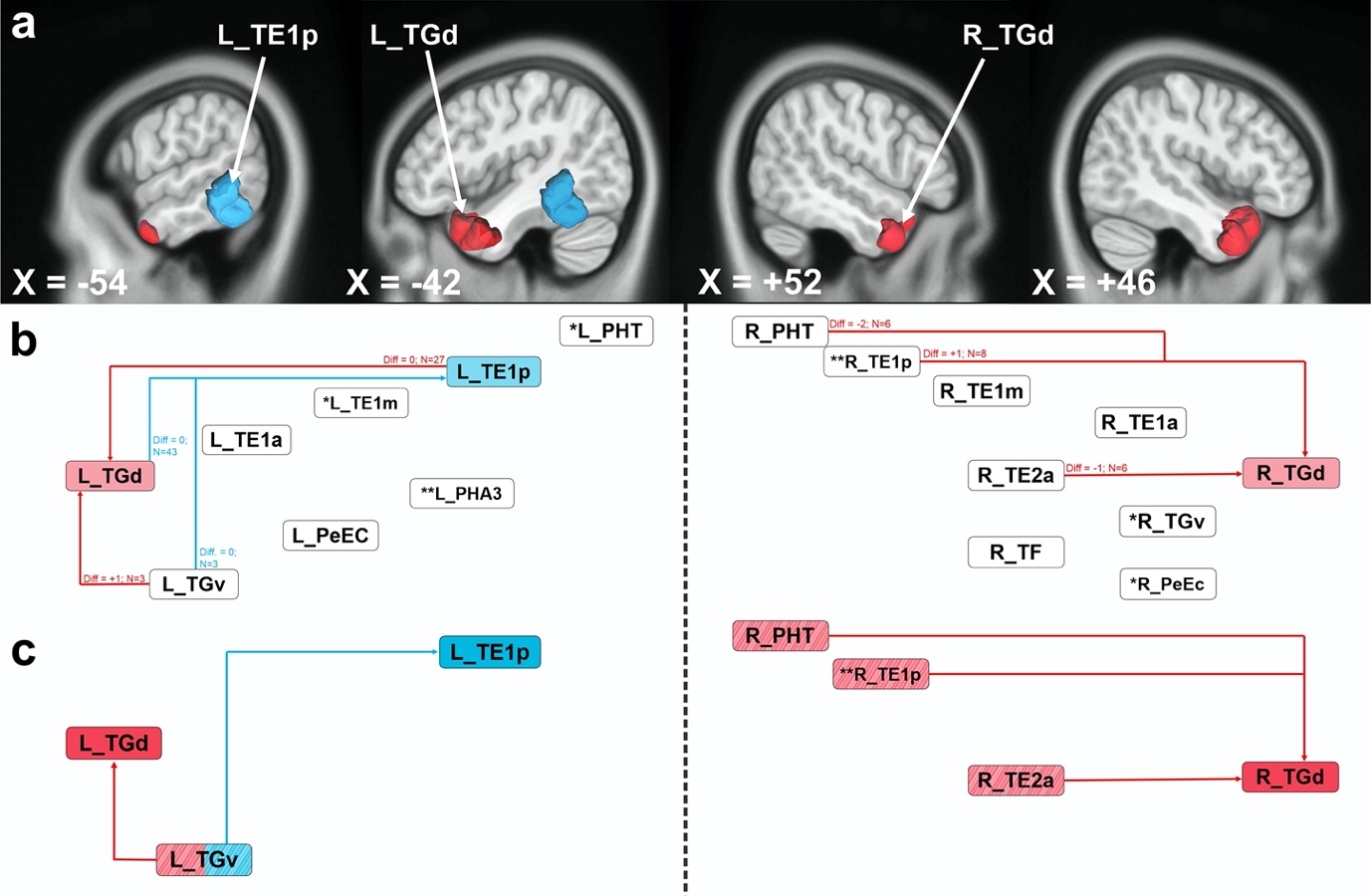


**SI Figure 5.** Sagittal sections from lateral to medial show the worst deletion areas which form the core of an epicenter for a specific connectotype, known as the epicenter parcellations, in the temporal lobe of the left (a) and right (b) hemispheres. A schematic diagram showing the relationship between epicenter parcellations and neighboring parcellations is shown for the left (c) and right (d) temporal lobes. A schematic diagram of varying connectotypes consisting of specific epicenters and neighboring parcellations in the left (e) and right (f) temporal lobes. Red: the most common epicenter in the respective lobe. Blue: the second most common epicenter in the respective lobe. Two colored rectangles: cortical areas with shared epicenters. Orange: areas with separate epicenters that could not be concluded with provided information. Asterisk: individual variability with different epicenters as shown. Diff: difference between the actual sequence and minimum path length between two connected areas. Minimal path length is referring to the minimum distance between two connected areas. Actual sequence is referring to the number of sequences for the connected epicenter to be shown in the deletion list. N: the number of individuals who have the respective worst area in deletion.

| **6. Temporal lobe** | | | |
| --- | --- | --- | --- |
| **Size of deletion** | | **Size of deletion** | |
| 1 | 43/80 L_TGd | 1 | 52/80 R_TGd |
| 2 | 23/80 **L_TGd**, L_PeEC | 2 | 27/80 R_TE2a, **R_TGd** |
| 3 | 43/80 **L_TGd**, L_TE2a, L_TE1p | 3 | 20/80 **R_TGd, R_TE2a**, R_TE1p |
| 4 | 29/80 **L_TGd, L_TE2a, L_PeEC, L_TE1p** | 4 | 29/80 **R_TGd, R_TE2a**, R_PHT, **R_TE1p** |
| 5 | 10/80 **L_TE1p, L_TGd**, L_TE1m, L_TE1a, **L_PeEC** | 5 | 12/80 **R_TE1p, R_TGd, R_TE2a, R_PHT**, R_TF |
| 6 | 7/80 **L_TE1p, L_TGd, L_TE1m, L_TE1a, L_PeEC**, L_TGv | | 12/80 **R_TE1p, R_TGd, R_TE2a, R_PHT**, R_PeEC |
|  | 7/80 L_PHA3, **L_TE1p**, L_TE2p, **L_TGd,** L_TF, **L_PeEC** | 6 | 10/80 **R_TE1p, R_TGd, R_TE2a, R_PHT, R_TF**, R_TGv |
| 7 | 8/80 **L_TE1p, L_TE2p, L_TGd, L_TE2a**, L_PHT, **L_TE1a, L_PeEC** | 7 | 11/80 **R_TE1p, R_TGd, R_TE2a, R_PHT, R_TF, R_PeEC, R_TGv** |
| 8 | 9/80 L_EC, **L_TE2p, L_TE1p, L_TGd, L_TE2a, L_TE1a, L_PeEC, L_TGv** | 8 | 17/80 **R_TE1p**, R_TE2p, **R_TGd, R_TE2a, R_PHT, R_TF, R_PeEC, R_TGv** |
| 9 | 18/80 **L_PHA3, L_TE1p, L_TE2p, L_TGd, L_TE1m, L_TE2a, L_TF, L_TE1a, L_PeEC** | 9 | 21/80 R_STSvp, **R_TE1p, R_TE2p, R_TGd, R_TE2a, R_PHT, R_TF, R_PeEC, R_TGv** |
| 10 | 33/80 **L_PHA3, L_TE1p, L_TE2p, L_TGd, L_TE1m,** L_PHA2, **L_TE2a, L_TF, L_TE1a, L_PeEC** | 10 | 33/80 L_PHA3, **L_TE1p, L_TE2p, L_TGd,** L_TE1m, L_PHA2, **L_TE2a**, **L_TF**, L_TE1a, **L_PeEC** |

**Supplementary Table S6.** List of the worst parcellation combinations deleted among 80 individuals in the temporal lobe. Parcellations that are bolded mean the occurrence of the worst deletion in previous combination. Progression of deleted combination has shown to be an accumulation of previous worst areas in percolation.

**Lateral parietal lobe**

Individuals demonstrated two unique connectotypes in the left hemisphere (6a) and three different connectotypes in the right hemisphere (6b) for the lateral parietal lobe. In the left lateral parietal lobe, L_7Am and L_PFm were the main epicenter parcellation of the left lateral parietal lobe, with 40 and 13 individuals sharing these common patterns, respectively. However, although the number of individuals with this characteristic was less significant, L_PGp and L_PFop could be a more significant epicenter than L_7Am in the lateral parietal lobe as the actual sequence for L_ 7Am to show up in the list is later than the expected distance. Therefore, for individuals with L_PGp and L_PFop as the worst areas, their epicenter progression starts from the anterior and lateral regions of the lateral parietal lobe. In the left lateral parietal lobe, L_PF, L_VIP, L_PGs and L_IPS1 belonged to L_7Am epicenter parcellation connectotype, while L_PFop, l_7AL, L_7Pm, L_MIP, L_Pgi and L_PGp belonged to connectotype with an epicenter around area L_PFm. L_7PC was observed to have a shared epicenter between L_7Am and L_PFm.

In the right lateral parietal lobe, R_PFm, R_ 7Am and R_7PC were the top worst areas in the right lateral parietal lobe, with 29, 15 and 10 individuals sharing these common patterns. Individuals with R_PGp and R_PF as the worst deletion have been shown to have different epicenters. R_PGp and R_PF have shown to be more closely related to an epicenter around R_ PFm than areas R_7Am and R_7PC. These results demonstrate that these subjects have epicenters located at the anterior and lateral regions of the lateral parietal lobe. Individuals with R_7PL as the worst deletions have different epicenters even though a limited individual had shown to share this characteristic for further interpretation. Furthermore, R_7PL, R_PGp, R_TPOJ3, R_7AL and R_PF belonged to R_PFm epicenter parcellation connectotype.


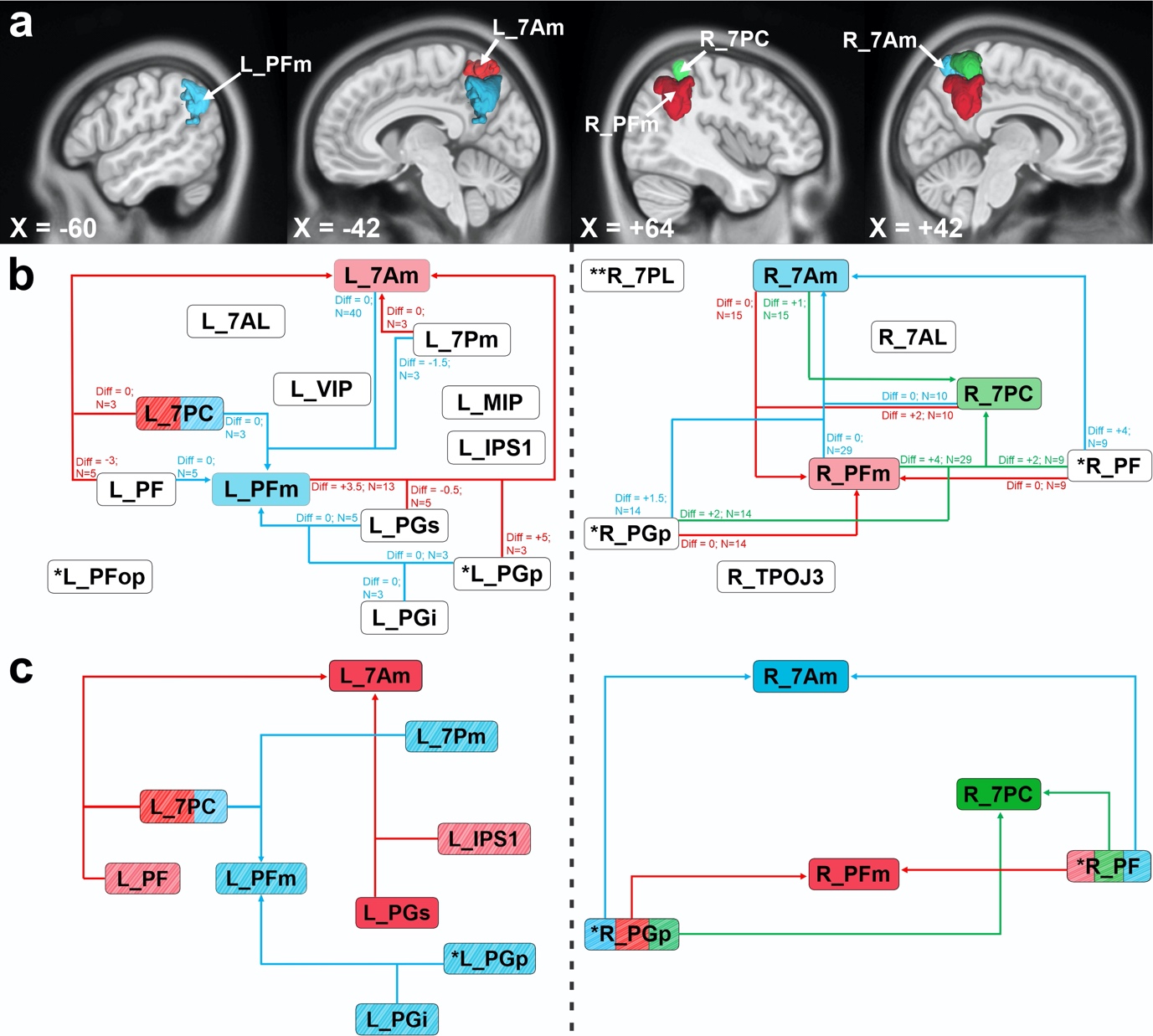
 **SI Figure 6.** Sagittal sections from lateral to medial show the worst deletion areas which form the core of an epicenter for a specific connectotype, known as the epicenter parcellations, in the lateral parietal lobe of the left (a) and right (b) hemispheres. A schematic diagram showing the relationship between epicenter parcellations and neighboring parcellations is shown for the left (c) and right (d) lateral parietal lobes. A schematic diagram of varying connectotypes consisting of specific epicenters and neighboring parcellations in the left (e) and right (f) lateral parietal lobes. Red: the most common epicenter in the respective lobe. Blue: the second most common epicenter in the respective lobe. Green: the third most common epicenter in the respective lobe. Two colored rectangles: cortical areas with shared epicenters. Asterisk: individual variability with different epicenters as shown. Diff: difference between the actual sequence and minimum path length between two connected areas. Minimal path length is referring to the minimum distance between two connected areas. Actual sequence is referring to the number of sequences for the connected epicenter to be shown in the deletion list. N: the number of individuals who have the respective worst area in deletion.

| **7. Lateral parietal lobe** | | | |
| --- | --- | --- | --- |
| **Size of deletion** | | **Size of deletion** | |
| 1 | 40/80 L_7AM | 1 | 29/80 R_PFm |
| 2 | 16/80 **L_7AM**, L_7PM | 2 | 27/80 R_PF, **R_PFm** |
| 3 | 11/80 L_PFm, L_PF, L_PGs | 3 | 19/80 **R_PFm**, R_PGp, R_PGs |
|  | 11/80 **L_PFm, L_PF**, L_PGi | 4 | 22/80 **R_PFm, R_PF, R_PGs, R_PGp** |
| 4 | 14/80 **L_PFm, L_PF, L_PGs, L_PGi** | 5 | 10/80 **R_PGs, R_PGp**, R_PGi, **R_PFm, R_PF** |
| 5 | 16/80 **L_PGs**, L_PGp, **L_PGi, L_PFm, L_PF** | 6 | 7/80 R_TPOJ2, **R_PGs, R_PGp, R_PGi, R_PFm**, R_TPOJ1 |
| 6 | 15/80 **L_PGs**, L_PFop, **L_PGp, L_PGi, L_PFm, L_PF** | 7 | 6/80 **R_TPOJ2, R_PGs**, R_TPOJ3, **R_PGp, R_PGi, R_PFm, R_PF** |
| 7 | 9/80 L_LIPv, L_VIP, L_7PC, L_IP2, **L_PFm, L_PF, L_7AM** | | 6/80 R_IP1, **R_PGs**, R_7PM, R_MIP, R_7PL, **R_PFm**, R_7AM |
| 8 | 13/80 **L_LIPv**, L_7AL, **L_7PC, L_VIP, L_IP2, L_PFm, L_PF, L_7AM** | 8 | 6/80 **R_IP1**, R_IP0, **R_PGs, R_MIP, R_PGp, R_PGi, R_PFm, R_PF** |
| 9 | 11/80 L_IP1, **L_LIPv, L_7AL, L_7PC, L_VIP, L_IP2, L_PFm, L_PF, L_7AM** | 9 | 12/80 **R_IP1**, R_LIPv, R_7AL, **R_PGs**, R_7PC, R_VIP, **R_MIP, R_PFm, R_7AM** |
| 10 | 10/80 **L_IP1, L_LIPv, L_7AL**, L_LIPd, **L_7PC, L_VIP, L_IP2, L_PFm, L_PF, L_7AM** | 10 | 18/80 **R_IP1, R_LIPv, R_7AL, R_PGs**, R_LIPd, **R_7PC, R_VIP, R_MIP, R_PFm, R_7AM** |

**Supplementary Table S7.** List of the worst parcellation combinations deleted among 80 individuals in the lateral parietal lobe. Parcellations that are bolded mean the occurrence of the worst deletion in previous combination. Progression of deleted combination has shown to be an accumulation of previous worst areas in percolation.

**Medial parietal lobe**

Individuals demonstrated two unique connectotypes in the left hemisphere (3a) and three different connecotypes in the right hemisphere (3b) for the medial frontal lobe. In the left medial parietal lobe, L_7m and L_POS2 were the main epicenter parcellations of the medial parietal lobe, with 39 and 11 individuals sharing these common patterns, respectively. Although the number of individuals with this characteristic was less significant, L_PCV and L_v23ab have a different epicenter. In a subset of individuals, L_PCV could be a more significant area than L_POS2 and L_7m in the medial parietal lobe as the sequence for L_POS2 and L_7m to show up in the list was later than the expected distance. For L_v23ab, the L_POS2 epicenter parcellation was less significant as it shows up in the deletion sequence later than the expected distance. In the left medial parietal lobe, L_DVT and L_POS1 belonged to the L_POS2 epicenter parcellation connectotype, while L_23d and L_v23ab belonged to L_7m epicenter parcellation connectotype. Furthermore, after grouping the cortical regions into epicenters, L_PCV and L_31a were observed to have a shared epicenter between L_POS2 and L_7m.

In the right medial parietal lobe, R_POS2, R_PCV and R_POS1 were the top worst areas in the right medial parietal lobe, with 42, 15 and 13 individuals sharing these common patterns, respectively. Individuals with R_DVT, R_31a and R_RSC as the worst deletions have been shown to have different epicenters. For individuals with R_DVT as the worst area, R_DVT was more closely related to epicenters around areas R_POS2 and R_POS1 than around the R_PCV epicenter. These results highlight that these subjects have epicenters that were located at the posterior region of the medial parietal lobe. Individuals with R_31a as the worst area were more closely related to epicenter around area R_POS2 than area R_PCV. R_RSC had shown to be more closely associated with the epicenter centered around R_POS1. Hence, individuals with R_31a and R_RSC as the worst area would have their epicenter progression originating from the posterior surface of the medial parietal lobe. After grouping the cortical regions into epicenters, R_23c and 31a belonged to same R_POS2 epicenter parcellation connectotype, while R_7m, R_RSC and R_DVT belonged to R_POS1 epicenter parcellation connectotype.


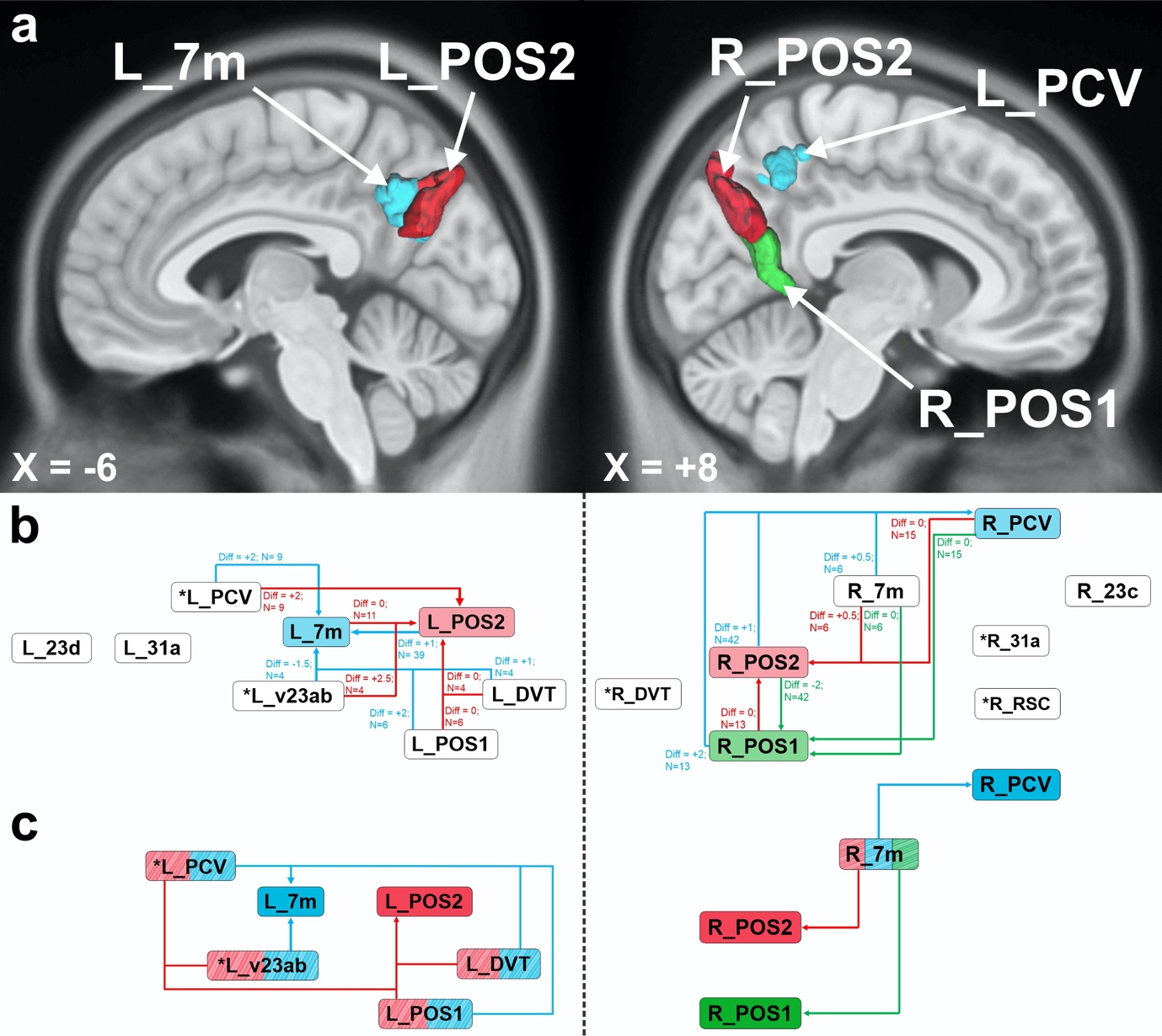


**SI Figure 7.** Sagittal sections show the worst deletion areas which form the core of an epicenter for a specific connectotype, known as the epicenter parcellations, in the medial parietal lobe of the left (a) and right (b) hemispheres. A schematic diagram showing the relationship between epicenter parcellations and neighboring parcellations is shown for the left (c) and right (d) medial parietal lobes. A schematic diagram of varying connectotypes consisting of specific epicenters and neighboring parcellations in the left (e) and right (f) medial parietal lobes. A schematic diagram of varying connectotypes consisting of specific epicenters and neighboring parcellations in the left (e) and right (f) medial parietal lobe. Red: the most common epicenter in the respective lobe. Blue: the second most common epicenter in the respective lobe. Green: the third most common epicenter in the respective lobe. Two colored rectangles: cortical areas with shared epicenters. Asterisk: individual variability with different epicenters as shown. Diff: difference between the actual sequence and minimum path length between two connected areas. Minimal path length is referring to the minimum distance between two connected areas. Actual sequence is referring to the number of sequences for the connected epicenter to be shown in the deletion list. N: the number of individuals who have the respective worst area in deletion. Red: the most common epicenter in the respective lobe. Blue: the second most common epicenter in the respective lobe. Green: the third most common epicenter in the respective lobe.

| **8. Medial parietal lobe** | | | |
| --- | --- | --- | --- |
| **Size of deletion** | | **Size of deletion** | |
| **1** | 39/80 L_POS2 | 1 | 42/80 R_POS2 |
| **2** | 24/80 L_7m, **L_POS2** | 2 | 33/80 **R_POS2**, R_POS1 |
| **3** | 34/80 L_POS1, L_RSC, **L_POS2** | 3 | 21/80 **R_POS2**, R_7m, R_PCV |
| **4** | 37/80 L_23c, **L_RSC**, L_23d, **L_POS1** | 4 | 26/80 **R_POS2, R_POS1, R_7m, R_PCV** |
| **5** | 20/80 L_31pv, L_31a, L_PCV, **L_23d, L_7m** | 5 | 11/80 **R_POS1, R_PCV, R_7m**, R_DVT, **R_POS2** |
| **6** | 18/80 L_d23ab, **L_31a, L_PCV, L_23d, L_7m, L_POS2** | 6 | 8/80 **R_POS1**, R_23c, **R_PCV, R_7m, R_DVT, R_POS2** |
| **7** | 31/80 **L_RSC, L_POS1, L_d23ab**, L_23c, **L_31a**, L_DVT, **L_POS2** | 7 | 10/80 **R_POS1, R_23c**, R_ProS, **R_PCV, R_7m, R_DVT, R_POS2** |
| **8** | 23/80 **L_POS1, L_d23ab**, L_ProS, **L_PCV, L_v23ab, L_7m**, L_31pd, **L_POS2** | 8 | 9/80 **R_POS1, R_23c**, R_31a, **R_ProS, R_PCV, R_7m, R_DVT, R_POS2** |
| **9** | 35/80 **L_POS1, L_d23ab, L_31pv, L_ProS, L_PCV**, L_v23ab, **L_7m, L_31pd, L_DVT** | 9 | 16/80 **R_POS1**, R_d23ab, **R_RSC, R_ProS, R_PCV**, R_v23ab, **R_7m, R_DVT, R_POS2** |
| **10** | 33/80 **L_POS1, L_d23ab, L_RSC, L_31pv, L_31a, L_v23ab, L_7m, L_31pd, L_DVT, L_POS2** | 10 | 28/80 **R_POS1, R_d23ab, R_RSC, R_23c, R_31a, R_PCV, R_v23ab, R_23d, R_7m, R_POS2** |

**Supplementary Table S8.** List of the worst parcellation combinations deleted among 80 individuals in the medial parietal lobe. Parcellations that are bolded mean the occurrence of the worst deletion in previous combination. Progression of deleted combination has shown to be an accumulation of previous worst areas in percolation.

**Occipital lobe**

Individuals demonstrated three unique connectotypes in the left hemisphere (3a) and three different connecotypes in the right hemisphere (3b) for the occipital lobe. In the left hemisphere, L_FFC, L_V2 and L_V3 were the main epicenter parcellations of the occipital lobe, which had 31, 21 and 14 individuals sharing these common patterns, respectively. Although the number of individuals with this characteristic was less significant, L_V1 and L_V2 could be a more significant area than L_FFC in the occipital lobe as the actual sequence for L_FFC to show up in the list was later than the expected distance. Therefore, for individuals with L_V1 and L_V2 as the worst areas, their epicenter progression likely starts from the posterior and medial regions of the occipital lobe. However, for individuals with L_PH as the worst area, L_V2 and L_V3 were less significant areas as compared to L_FFC. Therefore, for individuals with L_PH as the worst areas, their epicenter progression starts from the lateral region of the occipital lobe. After grouping the cortical regions into epicenters, the L_PH belonged to L_FFC epicenter parcellation connectotype, L_4 belonged to L_V3 epicenter and L_VVC belonged to L_V2 epicenter parcellation connectotype. For L_V1, it belonged to both L_V2 and L_V3 epicenter parcellation connectotypes in the medial and posterior surface of the left occipital lobe.

In the right occipital lobe, R_V1, R_FFC and R_V3 were the top worst areas in the right occipital lobe, with 14, 17 and 16 individuals sharing these common patterns. Individuals with R_V4 as the worst deletion have been shown to have different epicenters. R_V4 was shown to be more closely related to R_V1 and R_V3 epicenters than the R_FFC. This demonstrates that these subjects have their epicenters located at the posterior and lateral regions of the occipital lobe. Individuals with R_V3A and R_V7 as the worst deletions have different epicenters, even though limited individuals have been shown to share this characteristic for further interpretation. After grouping the cortical regions into epicenters, R_VVC, R_V7 and R_V4 belonged to R_V3 epicenter parcellation connectotype, while R_V2 belonged to the R_FFC epicenter parcellation connectotype. R_V3A belonged to both R_FFC and R_V3 epicenter parcellation connectotype.


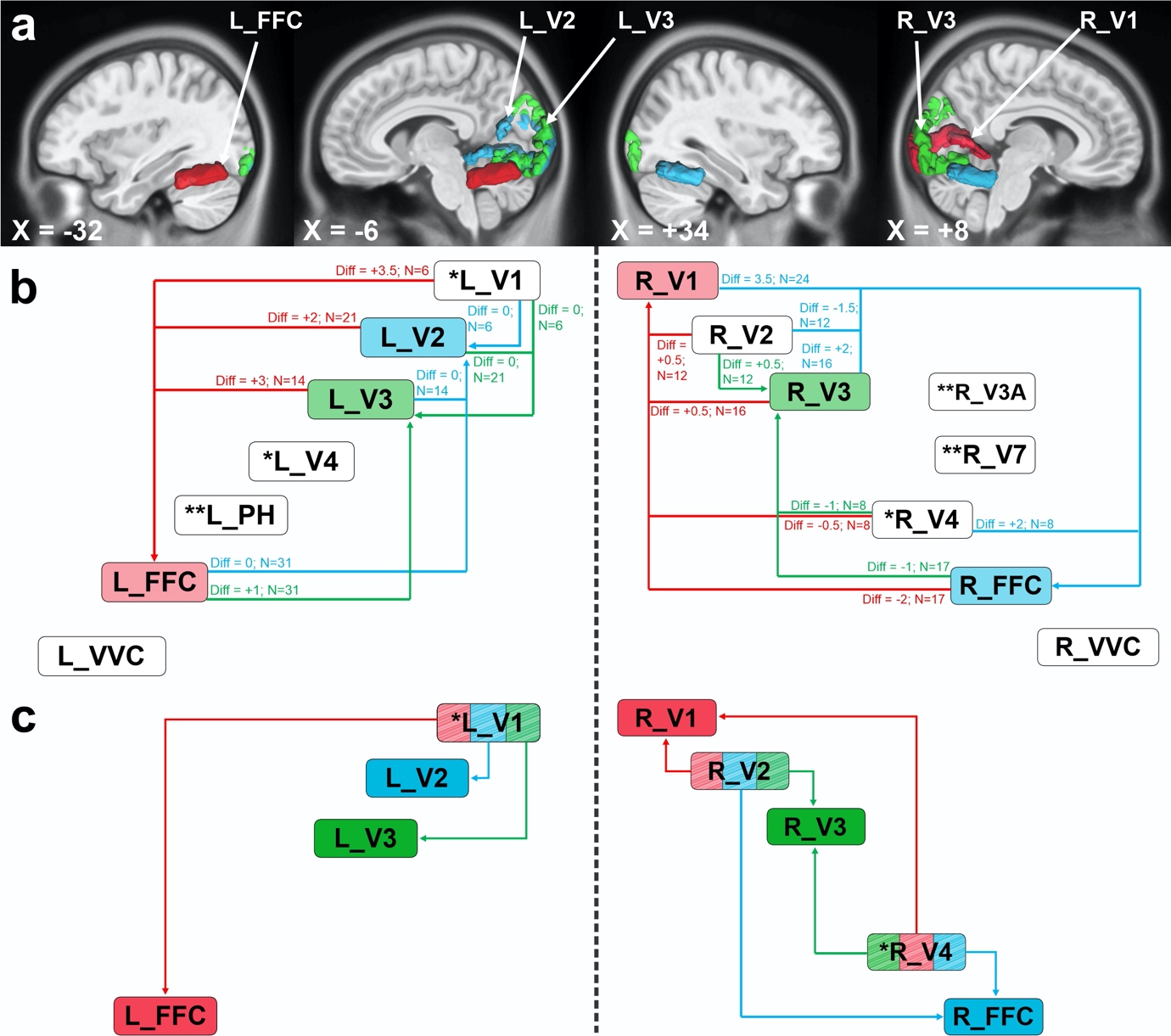


**SI Figure 8.** Sagittal sections from lateral to medial show the worst deletion areas which form the core of an epicenter for a specific connectotype, known as the epicenter parcellations, in the occipital lobe of the left (a) and right (b) hemispheres. A schematic diagram showing the relationship between epicenter parcellations and neighboring parcellations is shown for the left (c) and right (d) occipital lobes. A schematic diagram of varying connectotypes consisting of specific epicenters and neighboring parcellations in the left (e) and right (f) occipital lobes. Red: the most common epicenter in the respective lobe. Blue: the second most common epicenter in the respective lobe. Green: the third most common epicenter in the respective lobe. Two colored rectangles: cortical areas with shared epicenters. Asterisk: individual variability with different epicenters as shown. Diff: difference between the actual sequence and minimum path length between two connected areas. Minimal path length is referring to the minimum distance between two connected areas. Actual sequence is referring to the number of sequences for the connected epicenter to be shown in the deletion list. N: the number of individuals who have the respective worst area in deletion.

| **9. Occipital lobe** | | | |
| --- | --- | --- | --- |
| **Size of deletion** | | **Size of deletion** | |
| **1** | 31/80 L_FFC | 1 | 24/80 R_V1 |
| **2** | 27/80 **L_FFC**, L_VVC | 2 | 34/80 **R_V1**, R_V2 |
| **3** | 35/80 L_V1, L_V2, L_V3 | 3 | 48/80 **R_V1, R_V2**, R_V3 |
| **4** | 37/80 L_V4, **L_V1, L_V2, L_V3** | 4 | 52/80 R_V4, **R_V1, R_V2, R_V3** |
| **5** | 18/80 **L_V4, L_V2**, L_V8, **L_V3, L_FFC** | 5 | 26/80 **R_V4, R_V1, R_V2, R_V3**, R_PIT |
| **6** | 40/80 **L_V4, L_VVC, L_V2, L_V8, L_V3, L_FFC** | 6 | 18/80 **R_V4**, R_VVC, **R_V2**, R_V8, **R_V3**, R_FFC |
| **7** | 19/80 **L_V4**, L_V6, **L_VVC, L_V2, L_V8, L_V3, L_FFC** | 7 | 31/80 **R_V4, R_VVC, R_V2, R_V8, R_V3, R_PIT, R_FFC** |
| **8** | 23/80 **L_V4**, L_V3a, **L_V6, L_VVC, L_V2, L_V8, L_V3, L_FFC** | 8 | 30/80 **R_V4**, R_V3a, **R_VVC, R_V2, R_V8, R_V3, R_PIT, R_FFC** |
| **9** | 38/80 **L_V4, L_V3a, L_V6, L_VVC, L_V2, L_V8, L_V3**, L_PIT, **L_FFC** | 9 | 46/80 **R_V4, R_V3a**, R_VMV1, **R_VVC, R_V2, R_V8, R_V3, R_PIT, R_FFC** |

**Supplementary Table S9.** List of the worst parcellation combinations deleted among 80 individuals in the occipital lobe. Parcellations that are bolded mean the occurrence of the worst deletion in previous combination. Progression of deleted combination has shown to be an accumulation of previous worst areas in percolation.

**Supplementary Table S10.** The relationship between the parcellation and the epicenter for all 8 cortical regions analyzed per cerebral hemisphere. Min: minimal path length between two connected areas. Act: actual number for the connected epicenter to be shown in the deletion list. Diff: difference between the actual sequence and minimum path length between two connected areas. L, left; R, right.

| **10a. L_Frontal** | | | | | | | | | |
| --- | --- | --- | --- | --- | --- | --- | --- | --- | --- |
|  | **L_45** | | | **L_10d** | | | **L_8AV** | | |
|  | **Min** | **Act.** | **Diff.** | **Min** | **Act.** | **Diff.** | **Min** | **Act.** | **Diff.** |
| **L_45** | **/** | **/** | **/** | **4** | **4** | **0** | **4** | **3.5** | **0.5** |
| **L_10d** | **4** | **4** | **0** | **/** | **/** | **/** | **5** | **3** | **2** |
| **L_8AV** | **4** | **5** | **-1** | **5** | **5** | **0** | **/** | **/** | **/** |
| **L_a47r** | **2** | **4** | **-2** | **3** | **4** | **-1** | **4** | **3.5** | **0.5** |
| **L_44** | **2** | **2.5** | **-0.5** | **5** | **6** | **-1** | **3** | **3.5** | **-0.5** |
| **L_10pp** | **4** | **3** | **1** | **2** | **10** | **-8** | **3** | **3** | **0** |
| **L_9a** | **4** | **2** | **2** | **2** | **3** | **-1** | **3** | **/** | **/** |
| **L_8C** | **4** | **4** | **0** | **4** | **/** | **/** | **2** | **2** | **0** |
| **L_8BL** | **5** | **5** | **0** | **4** | **4** | **0** | **3** | **3** | **0** |
| **L_47l** | **2** | **3** | **-1** | **4** | **7** | **-3** | **6** | **4** | **2** |

| **10b R_Frontal** | | | | | | | | | | |
| --- | --- | --- | --- | --- | --- | --- | --- | --- | --- | --- |
|  | **R_8BL** | | | **R_8AV** | | | **R_44** | | |  |
|  | **Min** | **Act.** | **Diff.** | **Min** | **Act.** | **Diff.** | **Min** | **Act.** | **Diff.** |  |
| **R_8BL** | **/** | **/** | **/** | **3** | **3** | **0** | **5** | **3.5** | **1.5** |  |
| **R_8AV** | **3** | **3** | **0** | **/** | **/** | **/** | **3** | **3.5** | **-0.5** |  |
| **R_44** | **5** | **5.5** | **-0.5** | **3** | **4** | **-1** | **/** | **/** | **/** |  |
| **R_45** | **5** | **3** | **2** | **4** | **5** | **-1** | **2** | **7.5** | **-5.5** |  |
| **R_10d** | **4** | **4** | **0** | **5** | **4** | **1** | **5** | **4** | **1** |  |
| **R_a10p** | **5** | **2** | **3** | **5** | **3** | **2** | **5** | **4** | **1** |  |
| **R_a47r** | **5** | **6** | **-1** | **4** | **3** | **1** | **4** | **2** | **2** |  |
| **R_10pp** | **5** | **2** | **3** | **3** | **3** | **0** | **6** | **6** | **0** |  |
| **R_p47r** | **5** | **5** | **0** | **3** | **7** | **-4** | **3** | **2** | **1** |  |
| **R_p10p** | **4** | **3** | **1** | **4** | **/** | **/** | **5** | **/** | **/** |  |
| **R_IFSa** | **4** | **3** | **1** | **4** | **/** | **/** | **2** | **5** | **-3** |  |
| **R_i6-8** | **3** | **2** | **1** | **3** | **5** | **-2** | **5** | **3** | **2** |  |
| **R_47s** | **6** | **3** | **3** | **5** | **3** | **2** | **3** | **5** | **-2** |  |

| **10c Left sensorimotor cortex** | | | | | | |
| --- | --- | --- | --- | --- | --- | --- |
|  | **L_SFL** | | | **L_6ma** | | |
|  | **Min** | **Act.** | **Diff.** | **Min** | **Act.** | **Diff.** |
| **L_SFL** | **/** | **/** | **/** | **2** | **2** | **0** |
| **L_6ma** | **2** | **2** | **0** | **/** | **/** | **/** |
| **L_6mp** | **5** | **4** | **-1** | **2** | **4** | **2** |
| **L_5L** | **/** | **/** | **/** | **5** | **5** | **0** |
| **L_4** | **4** | **4** | **0** | **3** | **3.5** | **0.5** |
| **L_1** | **4** | **4** | **0** | **4** | **4** | **0** |
| **L_6d** | **4** | **5** | **1** | **3** | **4** | **1** |
| **L_55b** | **/** | **/** | **/** | **4** | **7** | **3** |

| **10d Right sensorimotor cortex** | | | | | | | | | |
| --- | --- | --- | --- | --- | --- | --- | --- | --- | --- |
|  | **R_SFL** | | | **R_4** | | | **R_6ma** | | |
|  | **Min** | **Act.** | **Diff.** | **Min** | **Act.** | **Diff.** | **Min** | **Act.** | **Diff.** |
| **R_SFL** | **/** | **/** | **/** | **2** | **4** | **2** | **4** | **2** | **-2** |
| **R_4** | **4** | **4** | **0** | **/** | **/** | **/** | **2** | **4** | **2** |
| **R_6ma** | **2** | **2** | **0** | **3** | **3** | **0** | **/** | **/** | **/** |
| **R_6d** | **4** | **4** | **0** | **3** | **2** | **-1** | **2** | **3.5** | **1.5** |
| **L_6v** | **5** | **5** | **0** | **4** | **2** | **-2** | **2** | **3.5** | **1.5** |
| **R_1** | **4** | **3** | **-1** | **2** | **4** | **2** | **4** | **2** | **-2** |
| **R_5L** | **5** | **2** | **-3** | **2** | **3.5** | **1.5** | **2** | **2** | **0** |

| **10e Left medial frontal lobe** | | | | | | |
| --- | --- | --- | --- | --- | --- | --- |
|  | **L_9m** | | | **L_SCEF** | | |
|  | **Min** | **Act.** | **Diff.** | **Min** | **Act.** | **Diff.** |
| **L_9m** | **3** | **3** | **0** | **/** | **/** | **/** |
| **L_SCEF** | **/** | **/** | **/** | **3** | **3** | **0** |
| **L_a24** | **2** | **2** | **0** | **3** | **3** | **0** |
| **L_24dd** | **4** | **3** | **-1** | **2** | **2** | **0** |
| **L_10v** | **4** | **4** | **0** | **5** | **2** | **-3** |
| **L_pOFC** | **4** | **5.5** | **1.5** | **6** | **2.5** | **-3.5** |

| **10f Right medial frontal lobe** | | | | | | | | | |
| --- | --- | --- | --- | --- | --- | --- | --- | --- | --- |
|  | **R_9m** | | | **R_10v** | | | **R_SCEF** | | |
|  | **Min** | **Act.** | **Diff.** | **Min** | **Act.** | **Diff.** | **Min** | **Act.** | **Diff.** |
| **R_9m** | **/** | **/** | **/** | **4** | **3** | **-1** | **3** | **4** | **1** |
| **R_10v** | **3** | **3** | **0** | **/** | **/** | **/** | **5** | **3** | **-2** |
| **R_SCEF** | **3** | **2** | **-1** | **8** | **5** | **-3** | **/** | **/** | **/** |
| **R_8BM** | **2** | **3.5** | **1.5** | **5** | **4** | **-1** | **2** | **2.5** | **0.5** |
| **R_24dd** | **4** | **6** | **2** | **8** | **6** | **-2** | **2** | **3** | **1** |
| **R_11l** | **5** | **3** | **-2** | **2** | **5.5** | **3.5** | **6** | **4** | **-2** |
| **R_13l** | **/** | **/** | **/** | **3** | **6** | **3** | **7** | **7** | **0** |
| **R_pOFC** | **6** | **2** | **-4** | **4** | **10** | **6** | **8** | **5** | **-3** |

| **10g Left operculum** | | | | | | |
| --- | --- | --- | --- | --- | --- | --- |
|  | **L_A4** | | | **L_Pol2** | | |
|  | **Min** | **Act.** | **Diff.** | **Min** | **Act.** | **Diff.** |
| **L_A4** | **/** | **/** | **/** | **3** | **2** | **-1** |
| **L_Pol2** | **2** | **4** | **2** | **/** | **/** | **/** |
| **L_PSL** | **2** | **2.5** | **0.5** | **/** | **/** | **/** |
| **L_OP4** | **3** | **2** | **-1** | **4** | **3** | **-1** |
| **L_43** | **2** | **2** | **0** | **/** | **/** | **/** |
| **L_MI** | **2** | **2** | **0** | **/** | **/** | **/** |
| **L_FOP4** | **3** | **2** | **-1** | **2** | **3** | **1** |
| **L_A5** | **2** | **2** | **0** | **3** | **4** | **1** |
| **L_AAIC** | **/** | **/** | **/** | **4** | **3** | **-1** |

| **10h Right operculum** | | | | | | | | | |
| --- | --- | --- | --- | --- | --- | --- | --- | --- | --- |
|  | **R_A4** | | | **R_43*** | | | **R_Pol2** | | |
|  | **Min** | **Act.** | **Diff.** | **Min** | **Act.** | **Diff.** | **Min** | **Act.** | **Diff.** |
| **R_A4** | **/** | **/** | **/** | **2** | **3** | **1** | **/** | **/** | **/** |
| **R_43*** | **/** | **/** | **/** | **/** | **/** | **/** | **/** | **/** | **/** |
| **R_Pol2** | **3** | **2** | **-1** | **/** | **/** | **/** | **2** | **3** | **1** |
| **R_PSL** | **2** | **2** | **0** | **/** | **/** | **/** | **/** | **/** | **/** |
| **R_OP4** | **3** | **2** | **-1** | **/** | **/** | **/** | **/** | **/** | **/** |
| **R_AAIC** | **/** | **/** | **/** | **/** | **/** | **/** | **/** | **/** | **/** |
| **R_A5** | **2** | **2** | **0** | **/** | **/** | **/** | **/** | **/** | **/** |
| **R_STGa** | **3** | **3.5** | **0.5** | **/** | **/** | **/** | **/** | **/** | **/** |

| **10i Left temporal lobe** | | | | | | |
| --- | --- | --- | --- | --- | --- | --- |
|  | **L_TGd** | | | **L_TE1p** | | |
|  | **Min** | **Act.** | **Diff.** | **Min** | **Act.** | **Diff.** |
| **L_TGd** | **/** | **/** | **/** | **3** | **3** | **0** |
| **L_TE1p** | **3** | **3** | **0** | **/** | **/** | **/** |
| **L_TE1a** | **2** | **2** | **0** | **3** | **2** | **-1** |
| ***L_TE1m** | **3** | **5** | **2** | **2** | **2** | **0** |
| ***L_PHT** | **4** | **5** | **1** | **2** | **2** | **0** |
| **L_TGv** | **2** | **3** | **1** | **3** | **3** | **0** |
| **L_PeEC** | **2** | **2** | **0** | **4** | **4** | **0** |
| ****L_PHA3** | **3** | **8** | **5** | **4** | **8** | **4** |

| **10j Right temporal lobe** | | | |
| --- | --- | --- | --- |
|  | **R_TGd** | | |
|  | **Min** | **Act.** | **Diff.** |
| **R_PHT** | **4** | **2** | **-2** |
| **R_TE1p** | **3** | **4** | **1** |
| **R_TE1m** | **3** | **3** | **0** |
| **R_TE1a** | **2** | **2** | **0** |
| **R_TE2a** | **3** | **2** | **-1** |
| **R_TF** | **3** | **2** | **-1** |
| ***R_TGv** | **2** | **7** | **5** |
| ***R_PeEc** | **2** | **6** | **4** |

| **10k Left lateral parietal lobe** | | | | | | |
| --- | --- | --- | --- | --- | --- | --- |
|  | **L_7Am** | |  | **L_PFm** |  |  |
|  | **Min** | **Act.** | **Diff.** | **Min** | **Act.** | **Diff.** |
| **L_7Am** | **/** | **/** | **/** | **5** | **4** | **-1** |
| **L_PFm** | **4** | **7** | **3** | **/** | **/** | **/** |
| **L_7AL** | **2** | **3** | **1** | **5** | **4** | **-1** |
| **L_7Pm** | **2** | **2** | **0** | **5** | **3.5** | **-1.5** |
| **L_7PC** | **3** | **3** | **0** | **4** | **4** | **0** |
| **L_VIP** | **2** | **2** | **0** | **4** | **7** | **3** |
| **L_MIP** | **/** | **/** | **/** | **3** | **3** | **0** |
| **L_IPS1** | **3** | **2** | **-1** | **4** | **8** | **4** |
| **L_PF** | **5** | **2** | **-3** | **2** | **2** | **0** |
| **L_PGs** | **5** | **4.5** | **-0.5** | **2** | **2** | **0** |
| **L_PFop** | **4** | **10** | **6** | **3** | **3** | **0** |
| **L_PGp** | **5** | **10** | **5** | **3** | **3** | **0** |
| **L_PGi** | **/** | **/** | **/** | **2** | **2** | **0** |

| **Left lateral parietal lobe** | | | | | | | | | |
| --- | --- | --- | --- | --- | --- | --- | --- | --- | --- |
|  | **R_PFm** | | | **R_7Am** | | | **R_7PC** | | |
|  | **Min** | **Act.** | **Diff.** | **Min** | **Act.** | **Diff.** | **Min** | **Act.** | **Diff.** |
| **R_PFm** | **/** | **/** | **/** | **5** | **5** | **0** | **4** | **8** | **4** |
| **R_7Am** | **5** | **5** | **0** | **/** | **/** | **/** | **3** | **4** | **1** |
| **R_7PC** | **4** | **6** | **2** | **3** | **3** | **0** | **/** | **/** | **/** |
| **R_7PL** | **4** | **2** | **-2** | **3** | **10** | **7** | **/** | **/** | **/** |
| **R_7AL** | **5** | **4** | **-1** | **2** | **2** | **0** | **2** | **3** | **1** |
| **R_PF** | **2** | **2** | **0** | **4** | **6** | **2** | **5** | **8** | **3** |
| **R_PGp** | **3** | **3** | **0** | **5** | **6.5** | **1.5** | **5** | **7** | **2** |
| **R_TPOJ3** | **3** | **4** | **1** | **/** | **/** | **/** | **/** | **/** | **/** |

| **10l Left medial parietal lobe** | | | | | | |
| --- | --- | --- | --- | --- | --- | --- |
|  | **L_POS2** | | | **L_7m** | | |
|  | **Min** | **Act.** | **Diff.** | **Min** | **Act.** | **Diff.** |
| **L_POS2** | **/** | **/** | **/** | **3** | **3** | **0** |
| **L_7m** | **2** | **3** | **1** | **/** | **/** | **/** |
| **L_23d** | **5** | **7** | **2** | **4** | **4** | **0** |
| **L_31a** | **4** | **4.5** | **0.5** | **3** | **2.5** | **-0.5** |
| **L_PCV** | **3** | **5** | **2** | **2** | **4** | **2** |
| **L_v23ab** | **3** | **5.5** | **2.5** | **4** | **2.5** | **-1.5** |
| **L_POS1** | **2** | **2** | **0** | **2** | **4** | **2** |
| **L_DVT** | **/** | **/** | **/** | **3** | **4** | **1** |

| **10m Right medial parietal lobe** | | | | | | |
| --- | --- | --- | --- | --- | --- | --- |
|  | **L_POS2** | | | **L_7m** | | |
|  | **Min** | **Act.** | **Diff.** | **Min** | **Act.** | **Diff.** |
| **L_POS2** | **/** | **/** | **/** | **3** | **3** | **0** |
| **L_7m** | **2** | **3** | **1** | **/** | **/** | **/** |
| **L_23d** | **5** | **7** | **2** | **4** | **4** | **0** |
| **L_31a** | **4** | **4.5** | **0.5** | **3** | **2.5** | **-0.5** |
| **L_PCV** | **3** | **5** | **2** | **2** | **4** | **2** |
| **L_v23ab** | **3** | **5.5** | **2.5** | **4** | **2.5** | **-1.5** |
| **L_POS1** | **2** | **2** | **0** | **2** | **4** | **2** |
| **L_DVT** | **/** | **/** | **/** | **3** | **4** | **1** |

| **10n Left occipital lobe** | | | | | | | | | |
| --- | --- | --- | --- | --- | --- | --- | --- | --- | --- |
|  | **L_FFC** | | | **L_V2** | | | **L_V3** | | |
|  | **Min** | **Act.** | **Diff.** | **Min** | **Act.** | **Diff.** | **Min** | **Act.** | **Diff.** |
| **L_FFC** | **/** | **/** | **/** | **5** | **5** | **0** | **4** | **3** | **-1** |
| **L_V2** | **4** | **6** | **2** | **/** | **/** | **/** | **2** | **2** | **0** |
| **L_V3** | **4** | **7** | **3** | **2** | **2** | **0** | **/** | **/** | **/** |
| **L_V1** | **5** | **8.5** | **3.5** | **2** | **2** | **0** | **3** | **3** | **0** |
| **L_V4** | **3** | **7** | **4** | **3** | **3** | **0** | **3** | **2** | **-1** |
| **L_PH** | **2** | **2** | **0** | **/** | **/** | **/** | **4** | **6** | **2** |
| **L_VVC** | **2** | **2** | **0** | **5** | **3** | **-2** | **4** | **3** | **-1** |

| **10o Right occipital lobe** | | | | | | | | | |
| --- | --- | --- | --- | --- | --- | --- | --- | --- | --- |
|  | **R_V1** | | | **R_FFC** | | | **R_V3** | | |
|  | **Min** | **Act.** | **Diff.** | **Min** | **Act.** | **Diff.** | **Min** | **Act.** | **Diff.** |
| **R_V1** | **/** | **/** | **/** | **5** | **8.5** | **3.5** | **3** | **3** | **0** |
| **R_FFC** | **5** | **3** | **-2** | **/** | **/** | **/** | **4** | **3** | **-1** |
| **R_V3** | **3** | **3.5** | **4** | **6** | **2** |  | **/** | **/** | **/** |
| **R_V2** | **2** | **2.5** | **0.5** | **4** | **2.5** | **-1.5** | **2** | **2.5** | **0.5** |
| **R_V3A** | **/** | **/** | **/** | **4** | **5** | **1** | **2** | **3** | **1** |
| **R_V7** | **4** | **5** | **1** | **5** | **5** | **0** | **4** | **3** | **-1** |
| **R_V4** | **4** | **3.5** | **-0.5** | **3** | **5** | **2** | **3** | **2** | **-1** |
| **R_VVC** | **4** | **4** | **0** | **2** | **2** | **0** | **4** | **3** | **-1** |

Supplementary References

1. Drewes C, Sagberg LM, Jakola AS, Solheim O. Perioperative and Postoperative Quality of Life in Patients with Glioma–A Longitudinal Cohort Study. *World Neurosurgery*. 2018/09/01/ 2018;117:e465-e474. doi:<https://doi.org/10.1016/j.wneu.2018.06.052>

2. Dhandapani M, Gupta S, Mohanty M, Gupta SK, Dhandapani S. Trends in cognitive dysfunction following surgery for intracranial tumors. *Surg Neurol Int*. 2016;7(Suppl 7):S190-S195. doi:10.4103/2152-7806.179229

3. Herbet G, Moritz-Gasser S. Beyond Language: Mapping Cognition and Emotion. *Neurosurg Clin N Am*. Jan 2019;30(1):75-83. doi:10.1016/j.nec.2018.08.004

4. Rijnen SJM, Kaya G, Gehring K, et al. Cognitive functioning in patients with low-grade glioma: effects of hemispheric tumor location and surgical procedure. *J Neurosurg*. Nov 15 2019:1-12. doi:10.3171/2019.8.JNS191667

5. Kahn E, Lane M, Sagher O. Eloquent: history of a word's adoption into the neurosurgical lexicon. *J Neurosurg*. Dec 2017;127(6):1461-1466. doi:10.3171/2017.3.JNS17659

6. Mandonnet E, Cerliani L, Siuda-Krzywicka K, et al. A network-level approach of cognitive flexibility impairment after surgery of a right temporo-parietal glioma. *Neurochirurgie*. 2017;63(4):308-313.

7. Yeung JT, Taylor HM, Young IM, Nicholas PJ, Doyen S, Sughrue ME. Unexpected hubness: a proof-of-concept study of the human connectome using pagerank centrality and implications for intracerebral neurosurgery. *Journal of Neuro-Oncology*. 2021/01/01 2021;151(2):249-256. doi:10.1007/s11060-020-03659-6

8. Chang EF, Kurteff G, Andrews JP, et al. Pure Apraxia of Speech After Resection Based in the Posterior Middle Frontal Gyrus. *Neurosurgery*. Sep 1 2020;87(3):E383-E389. doi:10.1093/neuros/nyaa002

9. Duffau H, Moritz-Gasser S, Mandonnet E. A re-examination of neural basis of language processing: proposal of a dynamic hodotopical model from data provided by brain stimulation mapping during picture naming. *Brain Lang*. Apr 2014;131:1-10. doi:10.1016/j.bandl.2013.05.011

10. Dadario NB, Brahimaj B, Yeung J, Sughrue ME. Reducing the Cognitive Footprint of Brain Tumor Surgery. *Front Neurol*. 2021;12:711646. doi:10.3389/fneur.2021.711646

11. Glasser MF, Coalson TS, Robinson EC, et al. A multi-modal parcellation of human cerebral cortex. *Nature*. Aug 11 2016;536(7615):171-178. doi:10.1038/nature18933

12. Bullmore E, Sporns O. Complex brain networks: graph theoretical analysis of structural and functional systems. *Nat Rev Neurosci*. Mar 2009;10(3):186-98. doi:10.1038/nrn2575

13. Briggs RG, Conner AK, Baker CM, et al. A Connectomic Atlas of the Human Cerebrum-Chapter 18: The Connectional Anatomy of Human Brain Networks. *Oper Neurosurg (Hagerstown)*. Dec 1 2018;15(suppl_1):S470-s480. doi:10.1093/ons/opy272

14. Bullmore E, Sporns O. The economy of brain network organization. *Nat Rev Neurosci*. Apr 13 2012;13(5):336-49. doi:10.1038/nrn3214

15. Rubinov M, Sporns O. Complex network measures of brain connectivity: uses and interpretations. *Neuroimage*. Sep 2010;52(3):1059-69. doi:10.1016/j.neuroimage.2009.10.003

16. Latora V, Marchiori M. Efficient behavior of small-world networks. *Physical review letters*. 2001;87(19):198701.

17. Berlot R, Metzler-Baddeley C, Ikram MA, Jones DK, O’Sullivan MJ. Global Efficiency of Structural Networks Mediates Cognitive Control in Mild Cognitive Impairment. Original Research. *Frontiers in Aging Neuroscience*. 2016-December-15 2016;8(292)doi:10.3389/fnagi.2016.00292

18. De Vico Fallani F, Richiardi J, Chavez M, Achard S. Graph analysis of functional brain networks: practical issues in translational neuroscience. *Philos Trans R Soc Lond B Biol Sci*. Oct 5 2014;369(1653)doi:10.1098/rstb.2013.0521

19. Li Y, Liu Y, Li J, et al. Brain Anatomical Network and Intelligence. *PLOS Computational Biology*. 2009;5(5):e1000395. doi:10.1371/journal.pcbi.1000395

20. Fischer FU, Wolf D, Scheurich A, Fellgiebel A. Association of structural global brain network properties with intelligence in normal aging. *PloS one*. 2014;9(1):e86258.

21. Del Ferraro G, Moreno A, Min B, et al. Finding influential nodes for integration in brain networks using optimal percolation theory. *Nature Communications*. 2018/06/11 2018;9(1):2274. doi:10.1038/s41467-018-04718-3

22. Latora V, Marchiori M. Efficient behavior of small-world networks. *Phys Rev Lett*. Nov 5 2001;87(19):198701. doi:10.1103/PhysRevLett.87.198701

23. Alstott J, Breakspear M, Hagmann P, Cammoun L, Sporns O. Modeling the impact of lesions in the human brain. *PLoS Comput Biol*. Jun 2009;5(6):e1000408. doi:10.1371/journal.pcbi.1000408

24. van den Heuvel MP, Sporns O. A cross-disorder connectome landscape of brain dysconnectivity. *Nature Reviews Neuroscience*. 2019/07/01 2019;20(7):435-446. doi:10.1038/s41583-019-0177-6

25. Andrews JP, Arora T, Theodosopoulos P, Berger MS. Paramedian transparietal approach to a dominant hemisphere intraventricular meningioma: illustrative case. *Journal of Neurosurgery: Case Lessons*. 16 Aug. 2021 2021;2(7):CASE21292. doi:10.3171/CASE21292

26. Spetzler RF, Martin NA. A proposed grading system for arteriovenous malformations. *J Neurosurg*. Oct 1986;65(4):476-83. doi:10.3171/jns.1986.65.4.0476

27. Cikla U, Swanson KI, Tumturk A, et al. Microsurgical resection of tumors of the lateral and third ventricles: operative corridors for difficult-to-reach lesions. *Journal of neuro-oncology*. 2016;130(2):331-340. doi:10.1007/s11060-016-2126-9

28. Chang EF, Gabriel RA, Potts MB, Berger MS, Lawton MT. Supratentorial cavernous malformations in eloquent and deep locations: surgical approaches and outcomes: Clinical article. *Journal of Neurosurgery JNS*. 01 Mar. 2011 2011;114(3):814-827. doi:10.3171/2010.5.Jns091159

29. Anderson RC, Connolly ES, Jr., Ozduman K, et al. Clinicopathological Review: Giant Intraventricular Cavernous Malformation. *Neurosurgery*. 2003;53(2):374-379. doi:10.1227/01.Neu.0000073533.52727.E4

30. Morone F, Makse HA. Influence maximization in complex networks through optimal percolation. *Nature*. 2015/08/01 2015;524(7563):65-68. doi:10.1038/nature14604

31. Guo S, Chen X, Liu Y, Kang R, Liu T, Daqing L. Percolation Analysis of Brain Structural Network. *Frontiers in Physics*. 07/01 2021;9:698077. doi:10.3389/fphy.2021.698077

32. Ahsan SA, Chendeb K, Briggs RG, et al. Beyond eloquence and onto centrality: a new paradigm in planning supratentorial neurosurgery. *J Neurooncol*. Jan 2020;146(2):229-238. doi:10.1007/s11060-019-03327-4

33. Yeung JT, Taylor HM, Young IM, Nicholas PJ, Doyen S, Sughrue ME. Unexpected hubness: a proof-of-concept study of the human connectome using pagerank centrality and implications for intracerebral neurosurgery. *J Neurooncol*. Jan 2021;151(2):249-256. doi:10.1007/s11060-020-03659-6

34. Crossley NA, Mechelli A, Scott J, et al. The hubs of the human connectome are generally implicated in the anatomy of brain disorders. *Brain*. Aug 2014;137(Pt 8):2382-95. doi:10.1093/brain/awu132

35. Mandal AS, Romero-Garcia R, Hart MG, Suckling J. Genetic, cellular, and connectomic characterization of the brain regions commonly plagued by glioma. *Brain*. Dec 5 2020;143(11):3294-3307. doi:10.1093/brain/awaa277

36. Lamar M, Charlton R, Zhang A, Kumar A. Differential associations between types of verbal memory and prefrontal brain structure in healthy aging and late life depression. *Neuropsychologia*. 2012;50(8):1823-1829.

37. Bai F, Shu N, Yuan Y, et al. Topologically convergent and divergent structural connectivity patterns between patients with remitted geriatric depression and amnestic mild cognitive impairment. *Journal of Neuroscience*. 2012;32(12):4307-4318.

38. Wen W, Zhu W, He Y, et al. Discrete neuroanatomical networks are associated with specific cognitive abilities in old age. *The Journal of Neuroscience*. 2011;31(4):1204. doi:10.1523/JNEUROSCI.4085-10.2011

39. Rudie JD, Brown JA, Beck-Pancer D, et al. Altered functional and structural brain network organization in autism. *NeuroImage: Clinical*. 2013/01/01/ 2013;2:79-94. doi:<https://doi.org/10.1016/j.nicl.2012.11.006>

40. Shu N, Liang Y, Li H, et al. Disrupted topological organization in white matter structural networks in amnestic mild cognitive impairment: relationship to subtype. *Radiology*. 2012;265(2):518-527.

41. Reijmer YD, Leemans A, Caeyenberghs K, et al. Disruption of cerebral networks and cognitive impairment in Alzheimer disease. *Neurology*. Apr 9 2013;80(15):1370-7. doi:10.1212/WNL.0b013e31828c2ee5

42. Caeyenberghs K, Leemans A, Leunissen I, et al. Altered structural networks and executive deficits in traumatic brain injury patients. *Brain Struct Funct*. Jan 2014;219(1):193-209. doi:10.1007/s00429-012-0494-2

43. Lawrence AJ, Chung AW, Morris RG, Markus HS, Barrick TR. Structural network efficiency is associated with cognitive impairment in small-vessel disease. *Neurology*. Jul 22 2014;83(4):304-11. doi:10.1212/wnl.0000000000000612

44. Li M, Jiang P, Wu J, et al. Altered Brain Structural Networks in Patients with Brain Arteriovenous Malformations Located in Broca's Area. *Neural Plast*. 2020;2020:8886803-8886803. doi:10.1155/2020/8886803

45. Garyfallidis E, Brett M, Amirbekian B, et al. Dipy, a library for the analysis of diffusion MRI data. *Front Neuroinform*. 2014;8:8. doi:10.3389/fninf.2014.00008

46. Mirzaalian H, Ning L, Savadjiev P, et al. Inter-site and inter-scanner diffusion MRI data harmonization. *NeuroImage*. 2016;135:311-323.

47. Glasser MF, Coalson TS, Robinson EC, et al. A multi-modal parcellation of human cerebral cortex. *Nature*. 2016;536(7615):171-178.

48. Tarjan R. Depth-first search and linear graph algorithms. *SIAM journal on computing*. 1972;1(2):146-160.

49. McKinney W. *Data Structures for Statistical Computing in Python*. 2010:56-61.

50. Harris CR, Millman KJ, van der Walt SJ, et al. Array programming with NumPy. *Nature*. Sep 2020;585(7825):357-362. doi:10.1038/s41586-020-2649-2

51. Hagberg A, Swart P, S Chult D. Exploring network structure, dynamics, and function using networkx. presented at: Conference: SCIPY 08 ; August 21, 2008 ; Pasadena; 2008; United States. <https://www.osti.gov/biblio/960616>

<https://www.osti.gov/servlets/purl/960616> AC52-06NA25396 2021-02-12

52. Csardi G, Nepusz T. The igraph software package for complex network research. *InterJournal, complex systems*. 2006;1695(5):1-9.

53. Ahsan SA, Chendeb K, Briggs RG, et al. Beyond eloquence and onto centrality: a new paradigm in planning supratentorial neurosurgery. *Journal of neuro-oncology*. 2020;146(2):229-238.
